# Niclosamide improves cancer immunotherapy by modulating RNA-binding protein HuR-mediated PD-L1 signaling

**DOI:** 10.1186/s13578-023-01137-w

**Published:** 2023-10-17

**Authors:** Qi Zhang, Zhe Yang, Xinbao Hao, Lauren J. Dandreo, Lily He, Yuxia Zhang, Fen Wang, Xiaoqing Wu, Liang Xu

**Affiliations:** 1 Department of Molecular Biosciences, The University of Kansas, 1567 Irving Hill Rd, Lawrence, KS 66045-7534 USA; 2 Department of Pharmacology, Toxicology & Therapeutics, The University of Kansas Medical Center, Kansas City, KS 66160 USA; 3 Department of Radiation Oncology, The University of Kansas Medical Center, Kansas City, KS 66160 USA; 4 The University of Kansas Cancer Center, The University of Kansas Medical Center, Kansas City, KS 66160 USA

**Keywords:** RNA-binding protein, HuR, PD-L1, Immunotherapy, Niclosamide, Immune evasion

## Abstract

**Background:**

Immune checkpoint blockade (ICB) represents a revolutionary advance in cancer treatment but remains limited success in triple-negative breast cancer (TNBC). Here we aim to explore the mechanism of RNA-binding protein (RBP) HuR in cancer immune evasion by post-transcriptionally regulating PD-L1 and evaluate the potential of HuR inhibition to improve immune response.

**Methods:**

The binding between HuR and PD-L1 mRNA was determined by ribonucleoprotein immunoprecipitation and RNA pull-down assays. The HuR knockout clones were established by CRISPR/Cas9 technology. The protein levels were assessed by Western blot, immunohistochemistry, and immunocytochemistry. The function and molecular mechanism of HuR-PD-L1 were determined by in vitro T cell activation and killing assay and in vivo efficacy assay.

**Results:**

We found that HuR directly bound to and stabilized PD-L1 mRNA. Knocking out HuR reduced PD-L1 levels and promoted T cell activation. We discovered that niclosamide reduced PD-L1 by inhibiting HuR cytoplasmic translocation, and diminished glycosylation of PD-L1. Niclosamide enhanced T cell-mediated killing of cancer cells and significantly improved the efficacy of anti-PD-1 immunotherapy in two syngeneic animal tumor models.

**Conclusion:**

We identified HuR as a novel posttranscriptional regulator of PD-L1, which plays an important role in tumor immune evasion. Niclosamide might be a promising repurposed drug to improve the patient response to immunotherapy by targeting HuR-PD-L1 axis. Our study demonstrates a novel strategy for targeting HuR/PD-L1 and provides the first proof-of-principle for repurposing niclosamide as a HuR inhibitor to overcome cancer immune evasion and improve response to ICB immunotherapy.

**Supplementary Information:**

The online version contains supplementary material available at 10.1186/s13578-023-01137-w.

## Introduction

Breast cancer (BC) has become the most common female cancer globally as of 2021, and triple-negative breast cancer (TNBC) accounts for about 10–15% of all breast cancer cases [1]. Currently, surgery, chemotherapy, and targeted therapy are the prevalent treatment paradigms for BC. Nevertheless, TNBC patients, unfortunately, have suffered from limited options and lack the benefits from targeted therapy, due to the loss of therapeutic targets, including estrogen receptor, progesterone receptor, and human epidermal growth factor receptor 2 [2].

Cancer immunotherapy with Immune checkpoint blockade (ICB), such as the antibodies against programmed cell death protein-1 (PD-1) and programmed death ligand-1 (PD-L1), has lately emerged as a revolutionary treatment strategy for various types of cancer [3–7], including melanoma and lung cancer, which exhibits a thrilling response rate of 50% [8, 9]. However, the overall response rate (ORR) of ICB varies across different solid tumors. Despite the long-term clinical benefits and curable potentials, the mean ORR of a single agent PD-1/PD-L1 antibody is only 19.56% in 91 clinical trials [10], and therapy resistance still remains a significant challenge for the application of PD-1/PD-L1 blockade. The ORR of TNBC patients to PD-1 antibody Pembrolizumab monotherapy was 18.5% in the KEYNOTE-012 Phase 1b [11], 5.3% in KEYNOTE-086 Phase 2 [12], and 9.6% in KEYNOTE-119 Phase 3 [13] with acceptable safety profile. The putative mechanisms that lead to resistance can be driven by tumor-extrinsic or -intrinsic factors, including the MYC pathway, autophagy, immunogenic cytokines, and immunosuppressive cytokines or exosomes [14]. Considering the fact that tumor infiltration of CD8^+^ T cell is vastly limited [15], thus BC has been clinically considered poor immune-responsive or immunologically “cold” [16] and patients who are suffering from metastatic BC have a particularly low ORR to ICB therapy, only from 3 to 21% [17]. Overall, it is an urgent and unmet medical need to overcome the BC immune evasion and improve BC response to ICB immunotherapy.

RNA-binding protein (RBP) Human antigen R (HuR), a member of the embryonic lethal abnormal vision-like (ELAVL) family, modulates the stability and translation of its target RNAs by binding to AU-rich elements (ARE) in their 3′-untranslated region (UTR) [18]. Among its targets, many protein-encoding-RNAs are implicated in a variety of biological processes and has been linked to numbers of diseases, including cancer. Hence, HuR plays an important role in multiple cancer hallmarks, including proliferation, metastasis, angiogenesis, and immune evasion. HuR is widely overexpressed in BC cells [19–22] and has been proved to be a promising therapeutic target to suppress tumor progression [23, 24]. More importantly, HuR had been reported to regulate the expression of immunosuppressive cytokine transforming growth factor beta (TGF-β) [25], which has been implicated in the tumor intrinsic mechanism of immune evasion. HuR also engaged in T cell activation via post-transcriptional regulation of immunogenic cytokines [26, 27]. Although these studies illustrate the underlying roles that HuR plays in tumor-associated immune evasion, currently no study has focused on the potential beneficial combination of restraining HuR and immune checkpoint genes.

Niclosamide is an anthelminthic drug approved by the United States Food and Drug Administration (FDA), presumably acting by the uncoupling of oxidative phosphorylation or stimulation of ATPase activity. In recent years, numerous studies have shown that niclosamide regulates multiple signaling pathways, including Wnt/β-catenin pathway, STAT3 pathway and NF-κB pathway [28]. These studies indicate that niclosamide may be developed as a novel treatment for many diseases beyond helminthic infection, especially cancer.

In this study, we found that HuR directly binds to 3′-UTR of *CD274* (PD-L1 mRNA) and elevated its half-life. Niclosamide was found to inhibit HuR cytoplasmic translocation, which prohibits HuR function in regulating mRNA turnover and translation. Knocking out HuR in MDA-MB-231 significantly enhanced T cell activation. Moreover, niclosamide disrupted glycosylation of PD-L1 and the accumulation of low glycosylated (LG) protein then triggered endoplasmic reticulum (ER) stress. Lastly, in two murine syngeneic tumor models, niclosamide dramatically promoted efficacy of anti-PD-1 antibody and significantly improved animal survival in vivo. Therefore, we uncovered a novel function of HuR in BC immune evasion, and HuR inhibition improved the efficacy of anti-PD-1 therapy. Our findings identified a novel strategy to overcome BC immune resistance by turning immune “cold” tumor into “hot” and thus improve BC response to ICB immunotherapy.

## Methods and materials

### Chemicals and reagents

Chemicals, antibodies, and siRNAs were listed in Additional file 1: Table S1.

Niclosamide was prepared in dimethyl sulfoxide (DMSO) at a concentration of 10 mmol/L as a stock for cell treatment. Niclosamide was dissolved in PBS with 10% Tween-80 and 5% ethanol at 2 mg/mL for animal study.

### Cell culture

All cells were listed in Additional file 1: Table S2 and cultured followed by American Type Culture Collection (ATCC) instructions. All cell lines were either recently obtained or monitored by short tandem repeat profiling. RNA isolation and RT- Quantitative RCR.

Total RNA was extracted, and reverse transcribed into cDNA following the manufacturer’s instructions. The primers used were listed in Additional file 1: Table S3.

### Protein extraction and immunoblotting

Total protein extraction and Western blot was performed as we previously described [19]. Nuclear and cytoplasmic protein extraction following the manufacturer’s instruction.

### Ribonucleoprotein immunoprecipitation and RNA pulldown assay

Two assays were carried out as reported previously [19] with minor modifications. *CD274* and *CANX* oligo was listed in Additional file 1: Table S3.

### PD-L1 mRNA decay assay

MDA-MB-231 sgControl, HuR knockout clones and MDA-MB-231 with niclosamide treatment (1.0 µM) were treated with Actinomycin D (5 µg/mL) for indicated time. The transcript expression was determined by qPCR.

### Immunohistochemistry staining, immunofluorescence staining and microscopy imaging

Immunohistochemistry (IHC) and Immunofluorescence staining was performed using standard staining procedures as previously described [29, 30].

#### Jurkat cells co-culture IL-2 secretion expression

T cell activation measurement was performed as described [31].

### T cell-mediated tumor cell killing assay

Human peripheral blood mononuclear cells (PBMCs) were cultured and activated according to the manufacturer’s protocol. The experiments were performed with anti-human CD3 Ab, human IgG4 isotype control, anti-human PD-1 (Pembrolizumab). GFP-expressing MDA-MB-231 cells were pre-seeded and treated with niclosamide (0, 2.5 or 5.0 μM), IgG or PD-1 Ab and T cells were added and then incubated for 48 h. The ratio between activated T cells and cancer cells (E:T ratio) is 1:1. The survival of cancer cells were quantified by GFP reading using BioTek Synergy H4 Hybrid Reader.

### Animal study

Female BALB/c mice and C57BL/6 mice aged 4–6 weeks purchased from Charles River Laboratories were used for efficacy studies. 2 × 10^5^ EMT6 or LL/2 cells in 0.2 mL DMEM were inoculated to #2 mammary fat pad. Mice with best matched tumors were randomized into six groups and then treated with 20 mg/kg niclosamide or IgG/anti-PD-1 100 µg/injection or vehicle control via intraperitoneal injection. Niclosamide or vehicle control was administered five times per week for four weeks, antibodies was administered twice per week for four weeks. Tumor sizes were measured by a caliper twice a week. Tumor volume was calculated using the formula: (length × width^2^)/2, as described previously [30]. Animal care and experiments were performed in accordance with the protocols approved by the Institutional Animal Use and Care Committee at the University of Kansas.

### Statistical analysis

The GraphPad Prism 9.0 software was used for statistical analysis. The Kaplan–Meier method and the log-rank test were used to compare overall survival, defined as the time of patients from surgery until death. Error bars in boxplots represent minimum and maximum values. Error bars in bar charts represent standard deviation except tumor growth data were expressed as mean ± standard error (SD) or mean (SEM). Student’s t test, one-way analysis of variance (ANOVA) followed by Dunnett's multiple comparisons test and two-way ANOVA followed by Šídák's multiple comparisons test were employed to analyze the in vitro and in vivo data. The Kaplan–Meier method and the Log-rank (Mantel-Cox) test were also used to analyze overall survival. A threshold *p* < 0.05 was defined as statistical significance. Exact sample sizes and number of replicates were indicated in the figure legends.

## Results

### HuR contributes to immune evasion in TNBCs

**Fig. 1:**
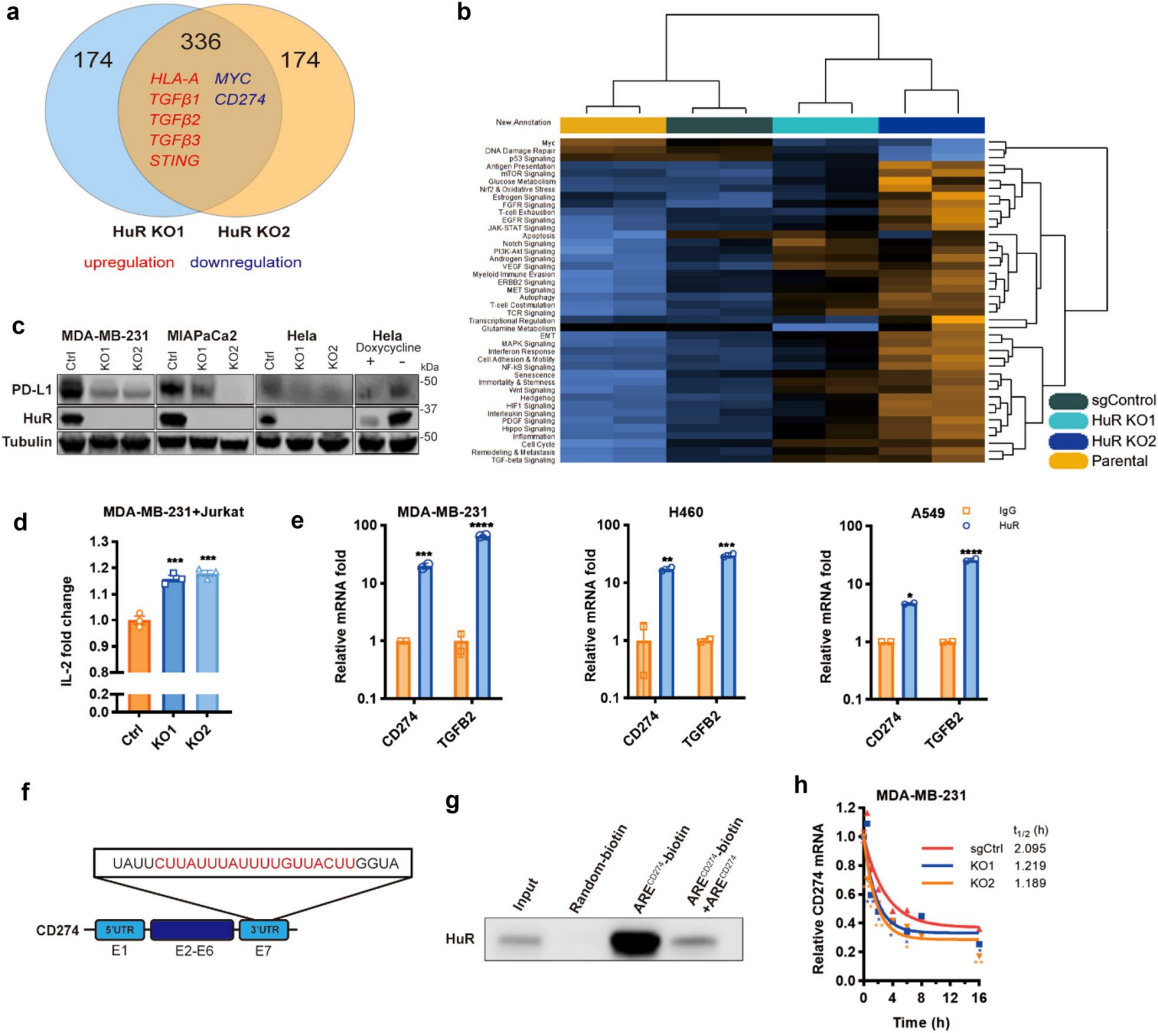
Identification of HuR as a regulator of PD-L1. **a** Venn diagram depicting the number of upregulated and downregulated targets identified in two HuR CRISPR/Cas9 knockout clones compared to sgCtrl. **b** Heatmap representation of enriched pathways in MDA-MB-231 parental cells, sgCtrl, and two HuR knockout clones. **c** Western blot analysis of HuR and PD-L1 protein level in HuR knockout MDA-MB-231, MIAPaCa2, HeLa cells, and doxycycline-inducible HuR Tet-off system in HeLa cells. **d** ELISA detection of IL-2 production in MDA-MB-231-Jurkat cell co-culture system (n = 3). **e** RNP-IP analysis of relative enrichment of PD-L1 transcripts in HuR-immunoprecipitation in MDA-MB-231, H460, and A549 cells (n = 2). f. Schematic representation of *CD274* (PD-L1 mRNA) structure. UTR, untranslated region; E, exon. *PD-L1* AREs sequences used for RNA pulldown assay were underlined in red. g. Representative Western blot of HuR protein in the pull-downed complex by *PD-L1* RNA probes in MDA-MB-231 cells. h. *PD-L1* mRNA decay in MDA-MB-231 cells at 0, 0.5, 1, 2, 4, 8, and 16 h post-treatment with 5 μg/mL of Actinomycin D. Two biological repeats for each group. Data are presented as mean ± SEM. One-way ANOVA (**d**). Two-way ANOVA (**e**). n.s.: no significance, *p < 0.05, **p < 0.01, ***p < 0.001, ****p < 0.0001

Traditionally, BC has been shown to be immunologically silent and hardly benefits from ICB. Though TNBC was suggested to be the most immunogenic subtype in BC due to the higher levels of lymphocyte infiltration, only a limited proportion of TNBC patients benefited from the enduring effect of ICB due to the resistance. In view of the limited understanding of ICB resistance in TNBC, we set out to study underlying mechanisms of immune evasion. HuR is highly expressed in TNBC and has been proved to function in metastasis, proliferation, and immune evasion. To further investigate the role of HuR in immune resistance, we manipulated the HuR expression in a human TNBC cell line MDA-MB-231 using CRISPR/Cas9 approach. Two stable HuR knockout (KO) clones were obtained as HuR KO1 and KO2. We then performed the Nanostring Gene set analysis using sgControl and these two HuR KO clones. The analysis results revealed a set of mRNAs changed in HuR KOs among total 510 genes (Fig. 1a), which were reported to participate in tumor immune resistance. And these two clones performed highly consistently among 42 pathways (Fig. 1b). Several immune evasion-related pathways including Autophagy, T cell costimulation, TCR signaling, and TGF-beta pathway were changed in HuR KOs (Additional file 1: Fig. S1a). These data indicate a potential role of HuR in TNBC immune evasion. HuR has been reported to be involved in PD-L1 regulation via CMTM6-PD-L1 axis [25]. Of note, the identification of AREs in the 3′-UTR of *CD274* were bound to Tristetraprolin (TTP), which exhibited competitive binding with HuR but had opposing effects on the bound mRNAs, provided us the rationale to test the potential binding between HuR and *CD274*. HuR-target interaction maps from CLIP-Seq on the starBase v2.0 (http://starbase.sysu.edu.cn/) were summarized in Additional file 1: Table S4, which revealed the binding between *CD274* and HuR in HEK293 and HUVEC cells [32].

We further examined PD-L1 protein levels in different cancer cell lines and that with HuR KO or over-expression. CRISPR/Cas9 induced KO of HuR in BC cell line MDA-MB-231 and pancreatic cancer cell line MIAPaCa2 led to significant reduction of PD-L1 protein levels in comparison with that of sgControl (Fig. 1c). Moreover, HuR deletion in cervical cancer HeLa cells also recapitulated the PD-L1 decrease, and over-expression in HeLa cells with HuR-Tet-off system increased PD-L1 (Fig. 1c). These data indicate that HuR regulates PD-L1. Since the interaction between tumor PD-L1 and PD-1 on T cell interferes with T cell activation and the acquisition of effector capacities, we next investigated whether HuR can influence PD-L1-mediated T cell suppression. IFN-γ-stimulated MDA-MB-231 sgRNA control or HuR-knockout cells were co-cultured with stimulated Jurkat cells for 48 h. The co-culture system was first validated (Additional file 1: Fig. S1b). HuR deficient cells secreted more IL-2 in comparison with that in the sgControl group (Fig. 1d). T cell killing ability was evaluated in the co-culture system using human PBMCs with MDA-MB-231 sgControl or HuR KO clones. Cell viability assay indicated that in HuR KO1, the T cell-mediated killing was improved compared to the sgControl (Additional file 1: Fig. S1c). These results collectively support that HuR plays a role in tumor immune evasion via regulating PD-L1 protein levels in TNBC.

### HuR binds directly to and stabilizes the PD-L1 mRNA

HuR is involved in post-transcriptional regulation of downstream mRNAs by binding to the ARE in the 3′-UTR of its target mRNAs [33]. To verify the interaction between PD-L1 mRNA transcript and HuR, ribonucleoprotein immunoprecipitation (RNP-IP) was performed in MDA-MB-231 and lung cancer cell lines H460 and A549. The qPCR results revealed the significant enrichment of *CD274* in HuR-immunoprecipitated complex in MDA-MB-231, H460 and A549 when compared with IgG control, thus validating the direct binding between *CD274* and HuR (Fig. 1e). We further carried out RNA-immunoprecipitation (RNA-IP) assay using the biotinylated *CD274* 3′-UTR RNA oligo to pull down bound HuR protein. Figure 1f shows the *CD274* gene schematic whereas ARE is marked in red. Figure 1g demonstrates that when compared with random RNA oligo, biotinylated ARE^CD274^ RNA shows enrichment of HuR. Moreover, unbiotinylated ARE^CD274^ RNA oligo could effectively compete with biotinylated RNA oligo for HuR binding and reduced the precipitated HuR. To explore the effect of HuR on *CD274* stability, we examined the *CD274* mRNA levels with or without HuR. As shown in Fig. 1h, *CD274* mRNA decay was expedited in the absence of HuR in MDA-MB-231 cells, further validated that HuR stabilized *CD274* mRNA. Taken together, our data demonstrate that HuR regulates *CD274* mRNA directly and promotes PD-L1.

## Niclosamide inhibits HuR function by blocking HuR cytoplasmic translocation, together with reduction of PD-L1

To identify potential inhibitors of HuR function, we set out screening assay targeting HuR expression and nucleo-cytoplasmic translocation. Consequently, the FDA-approved antihelmintic drug niclosamide was picked up as one of our top hits, and it has been reported to be a versatile multi-pathway inhibitor [34].

**Fig. 2:**
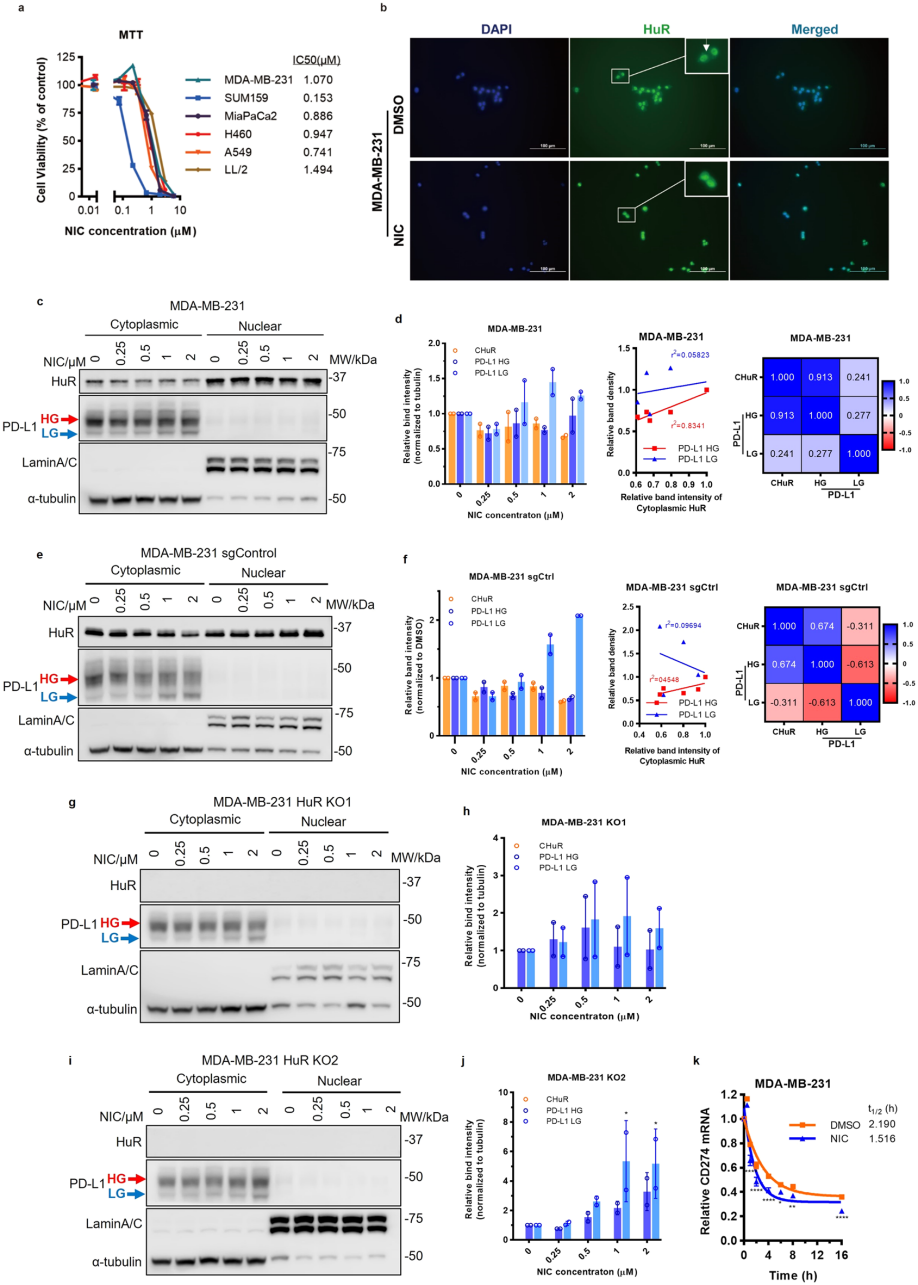
Niclosamide inhibits HuR nucleocytoplasmic shuttling. **a** MTT-based cytotoxicity assay of niclosamide on different cancer cells. **b** ICC staining of HuR in MDA-MB-231 cells treated with DMSO or 1 µM niclosamide (NIC) after 48 h. Utilizing DAPI stains cells nuclei blue. The white row indicates cytoplasmic HuR. Original magnification 40X; scale bar 100 µm. **c** Representative Western blot of HuR and PD-L1 protein in parental MDA-MB-231 cells. α-tubulin is used as the loading control. NIC, niclosamide. **d** Quantified relative level of cytoplasmic HuR in MDA-MB-231 cells (n = 2). Linear regression and correlation matrix between cytoplasmic HuR and PD-L1 relative band intensity. **e** Representative Western blot of HuR and PD-L1 protein levels in MDA-MB-231 sgCtrl cells. α-tubulin is used as the loading control. **f** Quantified relative level of HuR in MDA-MB-231 sgCtrl cells (n = 2). Linear regression and correlation matrix between cytoplasmic HuR and PD-L1 relative band intensity. Representative Western blot of HuR and PD-L1 protein levels in MDA-MB-231 HuR KO1 (**g**), and KO2 (**i**). Quantified relative level of cytoplasmic HuR and PD-L1 in MDA-MB-231 HuR KO1 (**h**), and KO2 (**j**) (n = 2). **k** *PD-L1* mRNA decay in MDA-MB-231 cells at 0, 0.5, 1, 2, 4, 8, and 16 h post-treatment with 1.0 μM niclosamide or DMSO and 5 μg/mL of Actinomycin D. Two biological repeats for each group. Data are presented as mean ± SEM. Two-way ANOVA (**d**, **f**, **h**, **j**, **k**). n.s.: no significance. Single linear regression and Pearson correlation coefficient (**d, f**). *p < 0.05, **p < 0.01, ***p < 0.001, ****p < 0.0001

Niclosamide exhibited potent cytotoxicity against a panel of cancer cell lines with the half maximal inhibitory concentration (IC50) 1.07 µM for MDA-MB-231 and 0.153 µM for SUM159 in MTT-based cytotoxicity assay (Fig. 2a**)**. To determine whether niclosamide affects HuR, we tested *ELAVL1* (HuR mRNA) and HuR total protein levels with niclosamide treatment at different concentrations or durations. However, we did not observe any significant changes in HuR mRNA or protein between control and treatment groups (Additional file 1: Fig. S2a–e). We then validated the effect of niclosamide on HuR nucleo-cytoplasmic translocation using immunocytochemistry (ICC) staining for HuR in cells treated with DMSO or niclosamide. As presented in Fig. 2b**,** HuR proteins were mainly restrained in nucleus after treatment with niclosamide in IC50 dose for 48 h. Various concentrations of niclosamide were applied to MDA-MB-231 cells for 16 h and nuclear and cytoplasmic fractions of the treated cells were isolated and immunoblotted. The cytoplasmic HuR levels decreased in a partial concentration-dependent manner. While examining PD-L1 protein levels in MDA-MB-231, we observed that the majority of PD-L1 was detected at approximately 45 kDa (the upper bind), while a small fraction was also detected at approximately 40 kDa (the lower band). To explore whether this pattern was associated with PD-L1 glycosylation, we treated MDA-MB-231 and its two HuR-knockout clones with tunicamycin to eliminate the entire N-glycan moiety and then tested the PD-L1 protein pattern with Western blot. The results showed that the majority of both the upper and lower bands was reduced to 33 kDa upon tunicamycin treatment in all cell lines (Additional file 1: Fig. S2f), suggesting that the two bands of PD-L1 with different molecular weights are different glycosylated forms. Accordingly, we labeled the upper band as high-glycosylated (HG) form, and the lower band as low-glycosylated (LG) form. Furthermore, niclosamide treatment results in reduction of HG PD-L1 as well as the cytoplasmic HuR levels (*r*^2^ = 0.8341, Fig. 2d). The Western blot relative band intensity, linear regression and correlation matrix were shown in Fig. 2c, d, respectively. Interestingly, niclosamide showed different effects on HuR translocation at high or low concentrations. At low concentration, niclosamide effectively inhibited HuR translocation, but at high concentration, 2 µM, the cytoplasmic HuR showed a certain amount of rebound, which might be due to the ER stress induced by niclosamide at higher concentration [35]. Overall, the above data support that niclosamide inhibits HuR function by effectively blocking HuR nucleo-cytoplasmic translocation.

As HuR plays an important role in PD-L1 regulation, we next investigated whether PD-L1 protein would be inhibited by niclosamide via blocking HuR translocation. sgControl and HuR-KO MDA-MB-231 cells were treated with different concentrations of niclosamide for 16 h. Niclosamide treatment reduced HG PD-L1 levels in a dose-dependent manner, consistent with its effect on cytoplasmic HuR (*r*^2^ = 0.4548) (Fig. 2e, f). However, in the absence of HuR, PD-L1 levels showed no obvious changes or even elevated as the niclosamide concentration increased (Fig. 2g–j). Compared with the decreased PD-L1 in the sgCtrl cells, these findings demonstrate that the niclosamide regulation of PD-L1 is, at least in part, HuR dependent. Given the identified interaction of HuR with *CD274*, we then aimed to investigate the effect of niclosamide on PD-L1 mRNA stability. The results showed that niclosamide treatment significantly decreased the half-life of PD-L1 mRNA (Fig. 2k). Together, these data suggest that niclosamide may act as a HuR translocation inhibitor, thereby promoting *PD-L1* mRNA decay.

## Niclosamide regulates PD-L1 glycosylation via ER stress-associated quality control

**Fig. 3:**
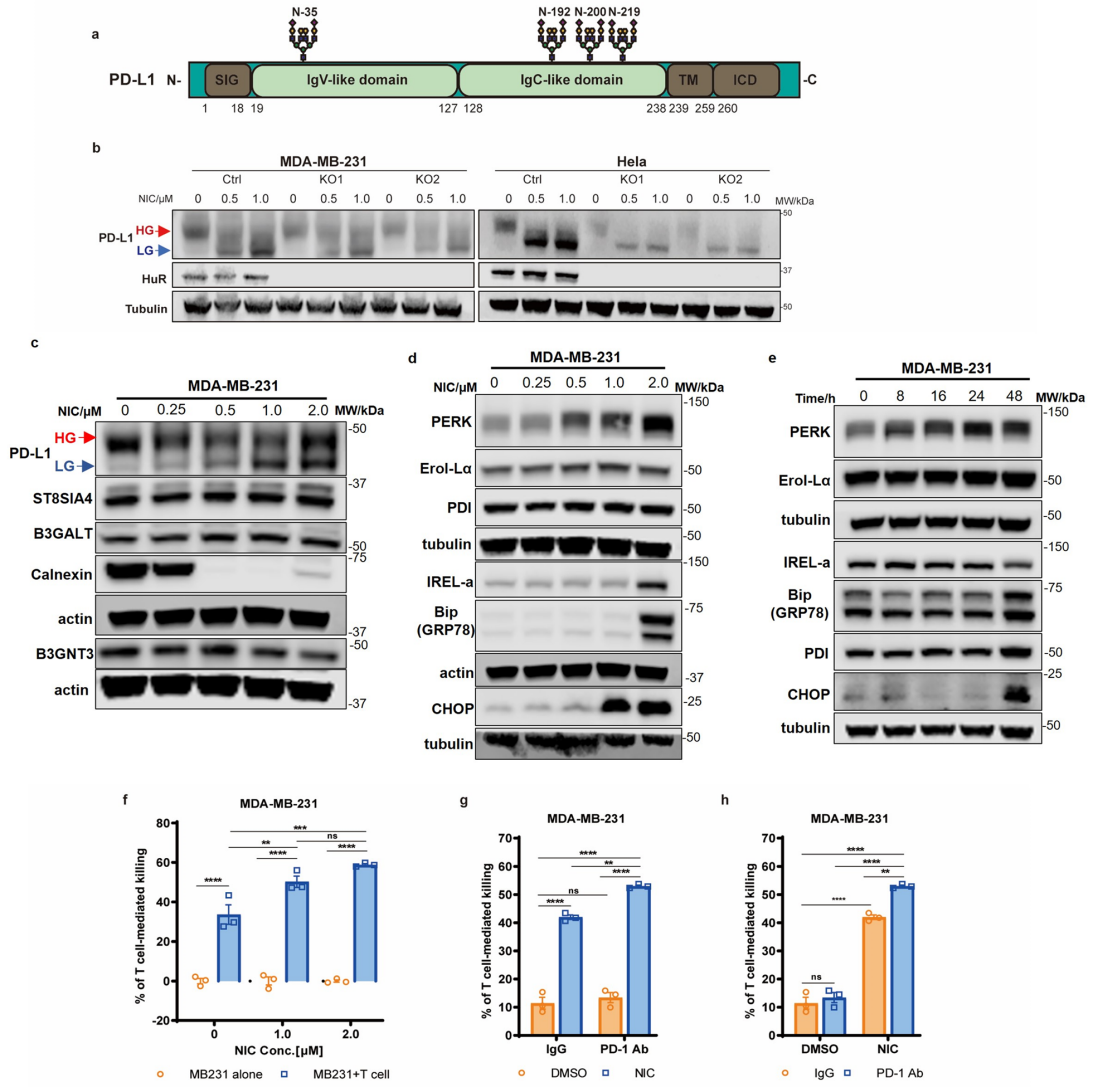
Niclosamide disrupts PD-L1 glycosylation and triggers ER stress. **a** The schematic of PD-L1 protein structure and glycosylation sites. SIG: signal peptide; IgV: immunoglobulin variable; IgC: immunoglobulin constant; TM: transmembrane; ICD: intracellular domain. **b** Representative Western blot of HuR and PD-L1 protein levels in sgControl and HuR-knockout MDA-MB-231 and HeLa cells. **c** PD-L1 and N-linked glycosylation associated protein levels upon niclosamide treatment in MDA-MB-231 cells. **d** Representative Western blot of multiple ER stress-related proteins in MDA-MB-231 cells with different concentrations of niclosamide treatment for 48 h. **e** Representative Western blot of multiple ER stress-related proteins in MDA-MB-231 cells with 1.0 µM niclosamide treatment for different time intervals. **f** GFP-expressing MDA-MB-231cells co-cultured with activated T cells for 48 h with or without niclosamide (1.0, 2.0 μM). The tumor cell to T cell ratio, 1:1 (n = 3). GFP- expressing MDA-MB-231 cells [38] co-cultured with activated T cell with or without niclosamide (**g**) and anti-PD-1 antibody (**h**). The tumor cell to T cell ratio, 1:1 (n = 3). The quantitative analysis of T cell-mediated killing is presented as mean ± SEM. Two-way ANOVA (**f**–**h**). ns, no significance, *p < 0.05, **p < 0.01, ***p < 0.001, ****p < 0.0001

N-linked glycosylation is one of the most important post-translational modifications of PD-L1, and the glycosylation site was demonstrated in schematic (Fig. 3a). Currently, several studies suggest that PD-L1 is highly glycosylated, and N-linked glycosylation plays an important role in PD-1/PD-L1-mediated tumor immunosuppression. The appropriate glycosylation of PD-L1 is essential for maintaining its protein stability and interaction with its receptor PD-1 [36]. Therefore, PD-L1 glycosylation is a potential therapeutic target to improve anti-PD-1/PD-L1 immunotherapy outcomes. The above results about PD-L1 glycosylation changes with niclosamide treatment prompted us to further explore the role of niclosamide and HuR in PD-L1 glycosylation. To this end, sgControl and HuR-KO MDA-MB-231 and HeLa cells were treated with niclosamide for 48 h. Despite the lower total PD-L1 levels in HuR-KO cells, LG PD-L1 was increased by niclosamide in both sgControl and HuR-KO MDA-MB-231 cells as well as HeLa cells (Fig. 3b). This result suggests that niclosamide disrupts proper PD-L1 glycosylation, and this regulation is likely not dependent on HuR. We then investigated how niclosamide regulates PD-L1 protein glycosylation and stability. We treated DMSO-/NIC-treated cells with the proteasome inhibitor MG132 and found that PD-L1 glycosylation pattern changed in a similar trend with or without MG132 co-treatment in both MDA-MB-231 and HeLa cells (Additional file 1: Fig. S3a–d). These results suggest that the changes in PD-L1 glycosylation were not affected by the proteasome-mediated degradation.

Next, we explored the mechanism that contributed to niclosamide-induced PD-L1 glycosylation dysfunction. We first inspected several proteins involved in the N-linked glycosylation process, including B3GNT3, ST8SIA4, B3GALT, and Calnexin in MDA-MB-231. With the treatment of different concentrations of niclosamide, we found that the protein levels of B3GNT3, ST8SIA4, and B3GALT didn’t change significantly; however, we observed an obvious change in Calnexin protein (Fig. 3c). As a chaperone located in ER, Calnexin is characterized by assisting protein folding and quality control and specifically acts to retain misfolded N-linked glycoproteins [37]. The dose-dependent decrease of Calnexin implied that PD-L1 glycosylation trigged by niclosamide treatment might be related to ER stress. ER contains a strict protein quality control system for proofreading newly synthesized proteins. Dysfunction of ER glycoproteins quality control such as protein glycosylation, assembling, and folding will cause the perturbation of ER-associated functions and lead to the unfolded protein response (UPR). Since we previously noticed the accumulation of low-glycosylated PD-L1 after niclosamide treatment, we then asked whether this aggregation activates UPR and induces ER stress. To this end, several ER stress-related proteins were tested with the treatment of niclosamide in different concentrations. Under the high concentration of niclosamide, we noticed the activation of PERK, IRE1α, BiP (also known as GRP-78) and CHOP, which are expressed specifically under the background of ER-stress (Fig. 3d). Moreover, cells treated with 1 µM niclosamide at different time points showed an obvious increase of PERK and CHOP (Fig. 3e). Additionally, we unexpectedly found an ARE in the 3′-UTR of *Calnexin* mRNA (Additional file 1: Fig. S3e), which implied that *Calnexin* mRNA might be a target of HuR. RNP-IP results suggest the potential HuR regulation of *Calnexin* mRNA in MDA-MB-231 and H460 (Additional file 1: Fig. S3f, g). HuR knockdown using siRNA in MDA-MB-231 led to the decrease of Calnexin (Additional file 1: Fig. S3h, i), with which further elucidation is warranted. Besides, PERK, IRE1α, BiP and CHOP didn’t show significant changes under HuR knockdown (Additional file 1: Fig. S3f).

The clinical success of anti-PD-1/PD-L1 therapies consists of the potentiation of anti-tumor immunity. Based on niclosamide-associated protein level and glycosylation changes in PD-L1, a T cell-mediated killing assay was carried to test the effect of niclosamide on cytotoxic T lymphocyte (CTL) activity. As expected, niclosamide treatment significantly enhanced T cell-mediated cancer cell death (Fig. 3f). To evaluate the possibility that niclosamide could improve PD-1 blockade, we tested the impact of niclosamide treatment on anti-PD-1 therapeutic activity in MDA-MB-231 and T cell co-culture system. We observed that the combined niclosamide and anti-PD-1 antibody treatment showed improved T cell-mediated killing compared with niclosamide, anti-PD-1 antibody alone, or control (Fig. 3g). Together, our data support that niclosamide enhances anti-tumor immunity by reducing PD-L1 protein.

## Niclosamide combined with anti-PD-1 antibody exhibits enhanced anti-tumor efficacy

**Fig. 4:**
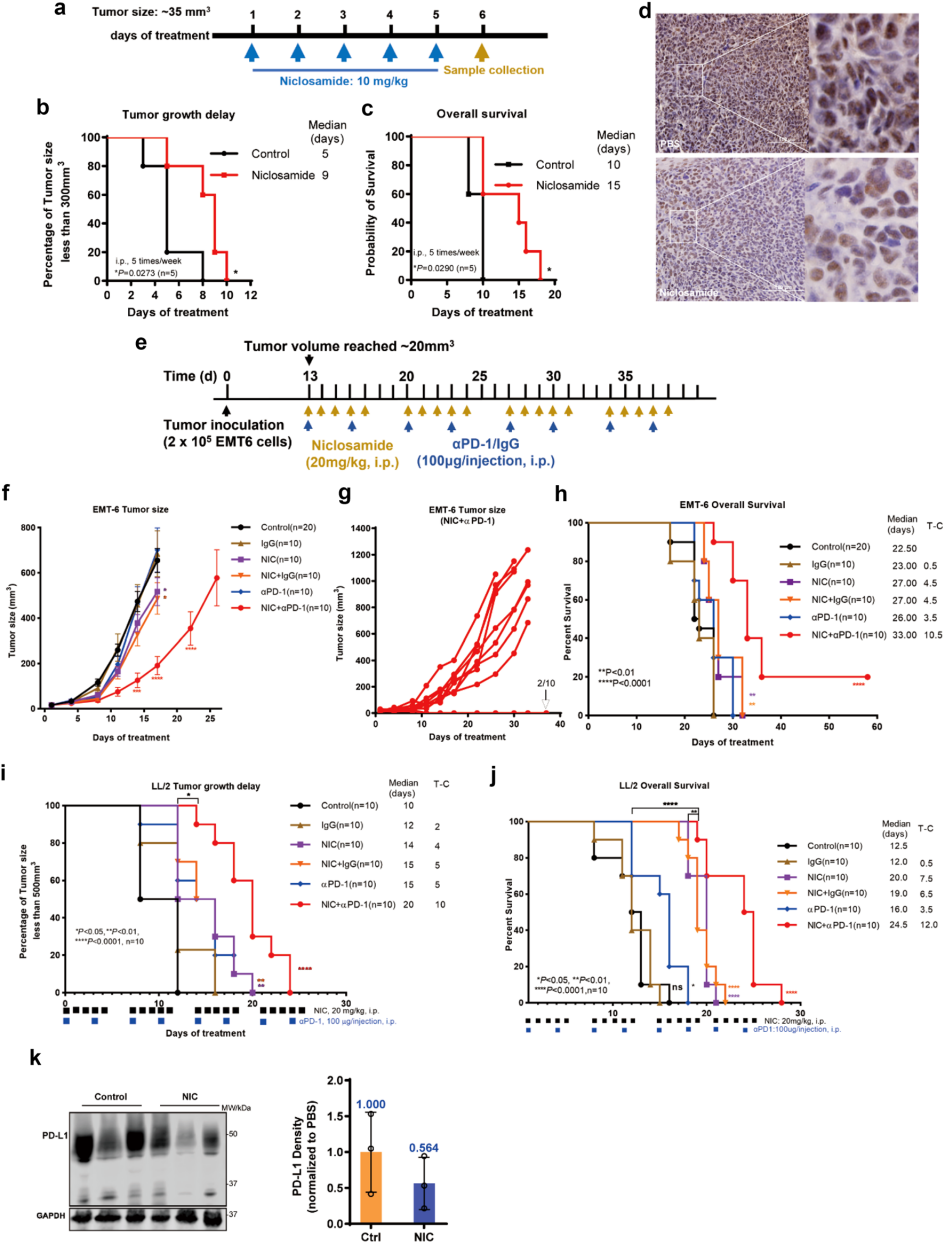
HuR inhibits T-cell activation in vitro and niclosamide potentiates anti-PD-1 efficacy in vivo*.* **a** Schematic illustrates the dosing regimens for mice bearing subcutaneous LL/2 tumors. **b** Tumor growth delay of LL/2 tumors under treatment with vehicle control or niclosamide. **c** Survival of mice bearing LL/2 tumors following treatment with vehicle control or niclosamide. Significance was determined by the log-rank test (n = 5 mice per group). **d** Representative images of IHC staining of HuR protein in LL/2 lung tumor samples treated with PBS control or niclosamide (10 mg/kg). **e** Schematic of animal experiment design in EMT-6 model. **f** tumor size of mice bearing EMT-6 tumors. **g** Individual tumor size of mice in the combination group. αPD-1, anti-PD-1 antibody. **h** Overall survival of mice bearing EMT-6 tumors. Kaplan–Meier curve of tumor growth delay (**i**) and overall survival (**j**) of mice bearing LL/2 tumors. **k** Representative PD-L1 protein immunoblots in EMT-6 tumor tissue samples and quantification of relative PD-L1 level (n = 3). Two-way ANOVA for tumor growth (**f**). Log-rank Mantel-Cox test for overall survival (**c**, **h**, and **j**) and tumor growth delay (**b** and **i**). n.s: no significance, *p < 0.05, **p < 0.01, ***p < 0.001, ****p < 0.0001

Results above suggest that niclosamide regulates PD-L1 through more than one mechanism, including inhibition of its upstream regulator HuR and protein glycosylation. To further examine the function of niclosamide in vivo, the tumor inhibition effect of niclosamide was tested in LL/2 mouse lung cancer model as shown in Fig. 4a. The tumor volumes were measured every day and shown in Additional file 1: Fig. S4a. The niclosamide group showed a significant inhibition in tumor growth (Fig. 4b) and a better survival rate (Fig. 4c) compared with the control group. In addition, consistent with the results in vitro, the IHC staining of HuR in the LL/2 tumors showed reduced cytoplasmic HuR after niclosamide treatment (Fig. 4d).

We then evaluated the efficacy of combining niclosamide and anti-PD-1 antibody in two different murine tumor models. In EMT-6 murine syngeneic breast cancer model, tumor-bearing female BALB/c mice were randomized into 6 groups and treated with either vehicle control, niclosamide (50 mg/kg via i.p. q.d.5), anti-PD-1 antibody (100 μg/injection via i.p. twice/week), or their combination for 4 weeks (Fig. 4e). Body weight and tumor sizes were measured routinely. No obvious side effects were noticed during the experiment. The mice in treatment group gained weight similar to those of control group (Additional file 1: Fig. S4b). Individual tumor size changes were shown in Additional file 1: Fig. S4c-g. The combination therapy significantly suppressed tumor growth (Fig. 4f) and impressively led to complete regression of EMT-6 tumors in 2 out of 10 mice (Fig. 4g). Niclosamide and PD-1 blockade together improved the animal survival, which was significantly better than either treatment alone (*p* < 0.001, n = 10) (Fig. 4h). The animal median survival time for the niclosamide, anti-PD-1 antibody and combination groups was 27, 26 and 33 days, respectively. We next tested this strategy using a different tumor model, the LL/2 murine syngeneic lung cancer model, to examine if our observed results are tumor type specific. We established the LL/2 model in C57BL/6 female mice and treated the mice similarly as described above. The body weight changes in treatment group were similar to those in the control group (Additional file 1: Fig. S5a). Individual tumor size changes were shown in Additional file 1: Fig. S5b-g. The niclosamide or anti-PD-1 antibody treatment inhibited tumor growth and supplementary animal survival, but the mice receiving niclosamide plus anti-PD-1 antibody combination treatment showed a more significant tumor growth delay (Fig. 4i) and significantly better overall survival (Fig. 4j), compared with that of either treatment alone (*p* < 0.001, n = 10). Additionally, EMT-6 tumor tissue Western blot data showed a reduction of PD-L1 protein upon niclosamide treatment (Fig. 4k).

**Fig. 5:**
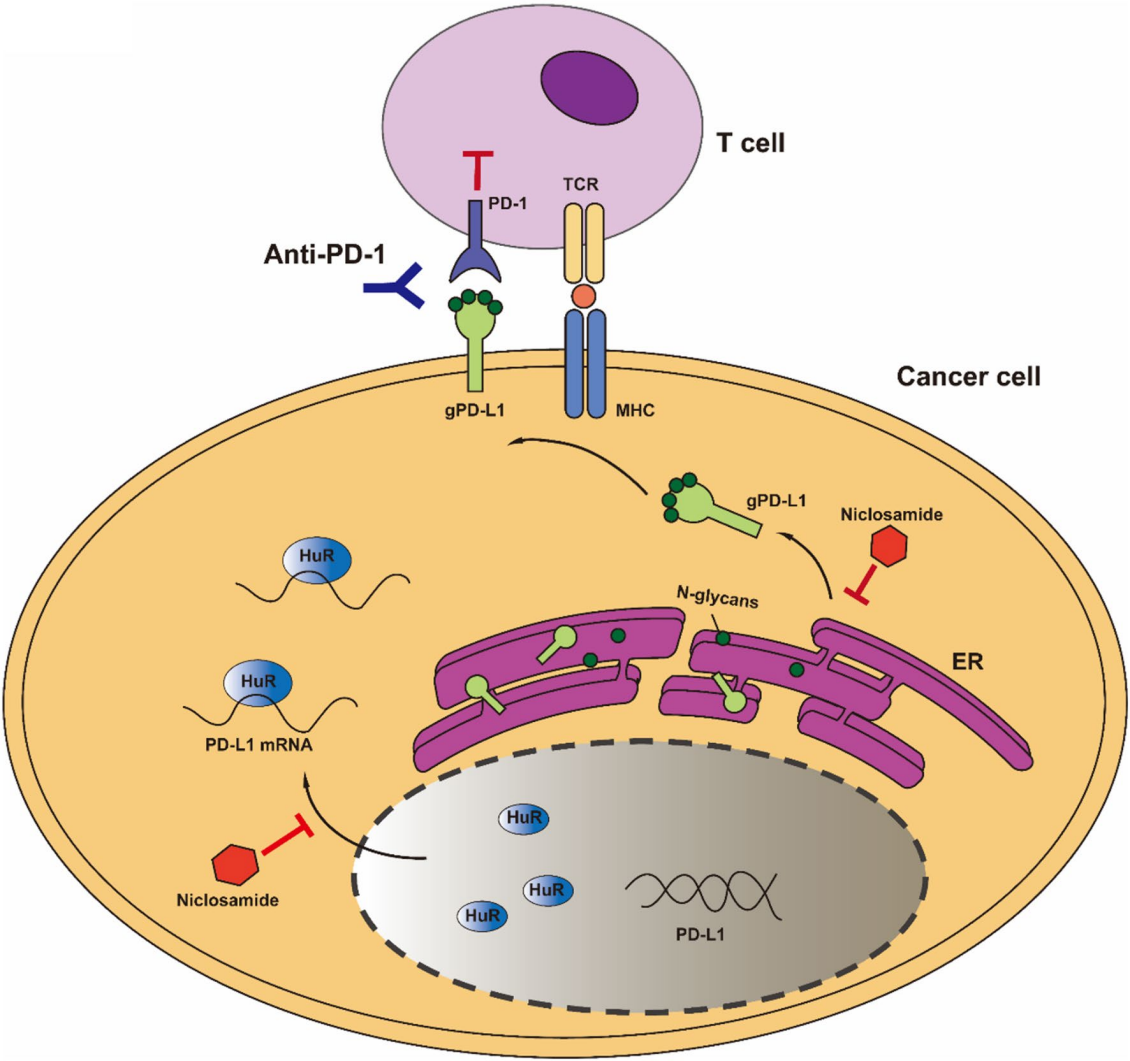
The proposed working model of niclosamide improves anti-PD-1 efficacy. Schematic diagram of the mechanism: niclosamide disrupts the nucleo-cytoplasmic shuttling of HuR, thereby inhibiting the posttranscription of *PD-L1* mRNA and restraining PD-1/PD-L1-mediated tumor immune evasion. In addition, niclosamide abolishes the glycosylation and maturation of PD-L1, thus leading to the decrease of functional PD-L1 form. Therefore, blocking HuR nucleocytoplasmic translocation and PD-L1 glycosylation by niclosamide can reverse the immunotherapy suppression and enhance anti-PD-1 efficacy

Taken together, our results demonstrate that the combination strategy using niclosamide plus anti-PD-1 antibody overcomes immunoresistance by improving T cell activity and improves the efficacy of ICB immunotherapy (Fig. 5, proposed working model).

## Discussion

A series of studies have dissected the putative molecular mechanisms underlying the ICB resistance in TNBC. High expression of immune checkpoint genes has been proved to be one of the immune evasion mechanisms. In this study, we discovered HuR as a novel regulator of immune checkpoint *PD-L1* mRNA in TNBC cells, which have a limited response to ICB targeting PD-1/PD-L1. Furthermore, we identified here, for the first time, niclosamide inhibits both HuR nucleo-cytoplasmic translocation and PD-L1 glycosylation that niclosamide can improve the anti-PD-1 immunotherapy efficacy in syngeneic murine breast and lung cancer models. Thus, our findings provide a new approach to improve anti-PD-1 immunotherapy in breast and lung tumors by repurposing niclosamide.

HuR has been reported to play an important role in cancer immune evasion by regulating TGF-β and cytokines [39, 40]. However, considering the complicated intrinsic mechanisms of ICB resistance in TNBC, the molecular mechanism driven by HuR has yet to be fully determined. Recently, Liu et al. described that HuR up-regulates PD-L1 expression on cancer cell surface via controlling *CMTM6* [25]. However, given the CLIP-seq data results of HuR binding sites in identified AREs in *CD274* 3′-UTR regulated by Tristetraprolin (TTP) [41, 42], their results cannot preclude the possibility that HuR was directly involved in PD-L1 regulation. In the current study, we identified HuR as a novel modulator of PD-L1 by directly binding to and stabilizing its mRNA. Additionally, we observed the function of HuR in impeding IL-2 secretion by T cells, which is crucial for T cell activation [43, 44]. These data enriched our understanding toward the roles of HuR in cancer immune evasion and PD-L1 regulation, suggesting a way for enhanced antitumor immunity by targeting HuR.

Drug repurposing has emerged as a promising strategy for developing anti-cancer drug, given the high cost and lengthy timeline of developing a new drug. Among those approved drugs, niclosamide has been revealed to exert a synergistic effect with numerous cancer drugs in human cancer cells. Besides the reported multi-mechanisms of niclosamide in Wnt/β-catenin signaling, NF-κB, mTORC1, STAT, or Notch pathways [28, 34], we report here a previously not yet recognized function of niclosamide: inhibiting HuR nucleocytoplasmic translocation, which was recently reported by our group in another study [45]. Interestingly, niclosamide shows different effects on HuR translocation at high or low concentrations, which may be attributed to the nature of HuR as a cell-stress responding protein, but the detailed underlying mechanism is still not fully understood. Yet, considering that niclosamide is not a HuR-specific inhibitor, this result indicates that niclosamide may have a broad inhibitive effect on other proteins that possesses nucleo-cytoplasmic shuttling activities. A previous study in NSCLC reported that niclosamide down-regulates PD-L1 expression by inhibiting p-STAT3 [46]. Considering the strong correlation between cytoplasmic HuR and HG PD-L1, we conclude that niclosamide reduces PD-L1 protein via inhibiting cytoplasmic HuR function. These findings not only uncover a new molecular mechanism by which niclosamide improves antitumor immunity, but also identify HuR as a novel post-transcriptional regulator of PD-L1. More in-depth analysis is warranted to better understand the multi-faceted roles of niclosamide in HuR-mediated PD-L1 regulation.

Besides the consistent downregulation of PD-L1 levels with niclosamide, we unexpectedly observed that niclosamide also has a potent effect on PD-L1 glycosylation profiling in various cancer cell lines. PD-L1 protein undergoes extensive post-translational modifications including phosphorylation, ubiquitination, acetylation, and glycosylation [47]. N-linked glycosylation is one of the most abundant post-translational modifications and contributes to various biological functions, including protein stability, ligand-receptor interaction, and subcellular localization [48]. The mechanisms of PD-L1 N-linked glycosylation via B3GNT3 [49], STT3 [50], and Sigma1 [51, 52] are studied in different cancer cells, and we didn’t detect changes of these proteins upon niclosamide treatment. However, we observed changes in several ER proteins following niclosamide treatment, indicating that ER stress may be a potential cause of PD-L1 glycosylation dysregulation. Considering the strong heterogeneity among cell signaling, clinical characteristics, and therapeutic responses in different cancer types, the niclosamide-induced PD-L1 glycosylation disorder is still worth elucidating.

Our experiments in murine breast and lung cancer models show that the combination of niclosamide and anti-PD-1 antibody had a synergetic effect compared with monotherapies. These results are consistent with the report from Fu et al. that inhibiting PD-L1 expression by niclosamide enhances PD-1/PD-L1 ICB. Our results also verified the strategy proposed by *Li, *et al. that targeting glycosylated PD-L1 to eradicate cancer cells, suggesting that niclosamide shows promise in improve immunotherapy efficacy through several pathways. Benefiting from the well-studied pharmacokinetics and pharmacodynamics and biological safety of niclosamide, repurposing of this drug can be attractive as the process is less risky, more cost-effective, and can be quickly moved into clinical testing. Currently, there are over 6 clinical trials testing niclosamide for its anti-tumor activity in various cancer populations (clinicaltrials.gov). With our findings of its new mechanism of action on HuR-PD-L1 signaling, it is worth redesigning the clinical trials to select patients with high levels of cytoplasmic HuR and PD-L1, who may have a better response to ICB immunotherapy. Other drugs with similar effect on HuR may also be explored for cancer immunotherapy.

Although this study sheds light on the roles of HuR in ICB resistance and the potential therapeutic value of niclosamide for TNBC patients, it has several limitations. Firstly, as a multifunctional drug, niclosamide has been reported to regulate multiple signaling pathways involved in cancer, so it is possible that niclosamide may also affect the other proteins involved in ICB resistance or immune evasion. Secondly, the precise molecular mechanisms underlying the interruption of PD-L1 glycosylation by niclosamide remain unclear. Further studies are necessary to identify the specific downstream targets of niclosamide in this context and elucidate the precise mechanisms by which it regulates PD-L1 glycosylation. Additional studies are needed to fully understand the potential off-target effects of niclosamide and its impact on other cellular processes.

In summary, we demonstrate a novel regulation mechanism of PD-L1 by HuR, which might be a promising new therapeutic target. The FDA-approved drug niclosamide, acting on both HuR translocation and PD-L1 glycosylation, may be a promising repurposed drug to overcome immune resistance to ICB and promote survival in vivo. Our study offers a strong proof-of-principle of repurposing niclosamide as the first HuR inhibitor to test in clinic, to modulate HuR-PD-L1 signaling to improve the response of ICB immunotherapy, especially for the immune “cold” breast cancer.

## Conclusion

HuR is a novel regulator of PD-L1. Niclosamide acting on both HuR translocation and PD-L1 glycosylation, might be a promising repurposed drug to overcome immunotherapy resistance and promote survival in vivo. This study reveals the role of HuR in tumor immune evasion and offers evidence of repurposing niclosamide as the first HuR inhibitor to modulate HuR-PD-L1 signaling and improve the immunotherapy response.

### Supplementary Information

**Additional file 1:** Supplementary file 1.

## Data Availability

The authors confirm that the data supporting the findings of this study are available within the article and/or its supplementary materials.

## Abbreviations

PD-L1: Programmed death-ligand 1
PD-1: Programmed cell death protein 1
RBP: RNA-binding protein
HuR: Human antigen R
FDA: Food and Drug Administration
NIC: Niclosamide
ICB: Immune checkpoint blockade
BC: Breast cancer
TNBC: Triple-negative breast cancer
CRISPR: Clustered regularly interspaced short palindromic repeats
Cas9: CRISPR-associated protein 9
ORR: Overall response rate
ARE: AU-rich elements
UTR: Untranslated region
TGF-β: Transforming growth factor-beta
STAT3: Signal transducer and activator of transcription 3
NF-κB: Nuclear factor kappa B
HG: High-glycosylated
LG: Low-glycosylated
IHC: Immunohistochemistry
ICC: Immunocytochemistry
LL/2: Lewis lung-2
PBMC: Peripheral blood mononuclear cell
KO: Knockout
TTP: Tristetraprolin
SD: Standard error
SEM: Standard error of the mean
ANOVA: Analysis of variance
CLIP-Seq: Cross-linking and immunoprecipitation
RNP-IP: Ribonucleoprotein immunoprecipitation
qPCR: Quantitative polymerase chain reaction
DMSO: Dimethyl sulfoxide
IFN-γ: Interferon gamma
ER: Endoplasmic Reticulum
B3GNT3: Beta-1,3-N-acetylglucosaminyltransferase
ST8SIA4: ST8 alpha-N-acetyl-neuraminide alpha-2,8-sialyltransferase 4
B3GALT: Beta-1,3-galactosyltransferase 5
UPR: Unfolded protein response
PERK: Protein kinase-like endoplasmic reticulum kinase
IRE1α: Inositol-requiring transmembrane kinase/endoribonuclease 1α
BiP: Binding immunoglobulin protein
GRP78: 78-KDa glucose-regulated protein
CHOP: The C/EBP Homologous Protein
CTL: Cytotoxic T lymphocyte
SIG: Signal peptide
IgV: Immunoglobulin variable
IgC: Immunoglobulin constant
TM: Transmembrane
ICD: Intracellular domain
mTORC1: Mechanistic target of rapamycin complex 1
