# Supplementary material for: Niclosamide improves cancer immunotherapy by modulating RNA-binding protein HuR-mediated PD-L1 signaling
Source: Cell Biosci. 2023 Oct 17;13:192. doi: 10.1186/s13578-023-01137-w (PMC10583380; doi:10.1186/s13578-023-01137-w)
Supplement: Supplementary file 1 — Additional file 1: Supplementary file 1. [file 13578_2023_1137_MOESM1_ESM.docx]

**Supplementary data**

**
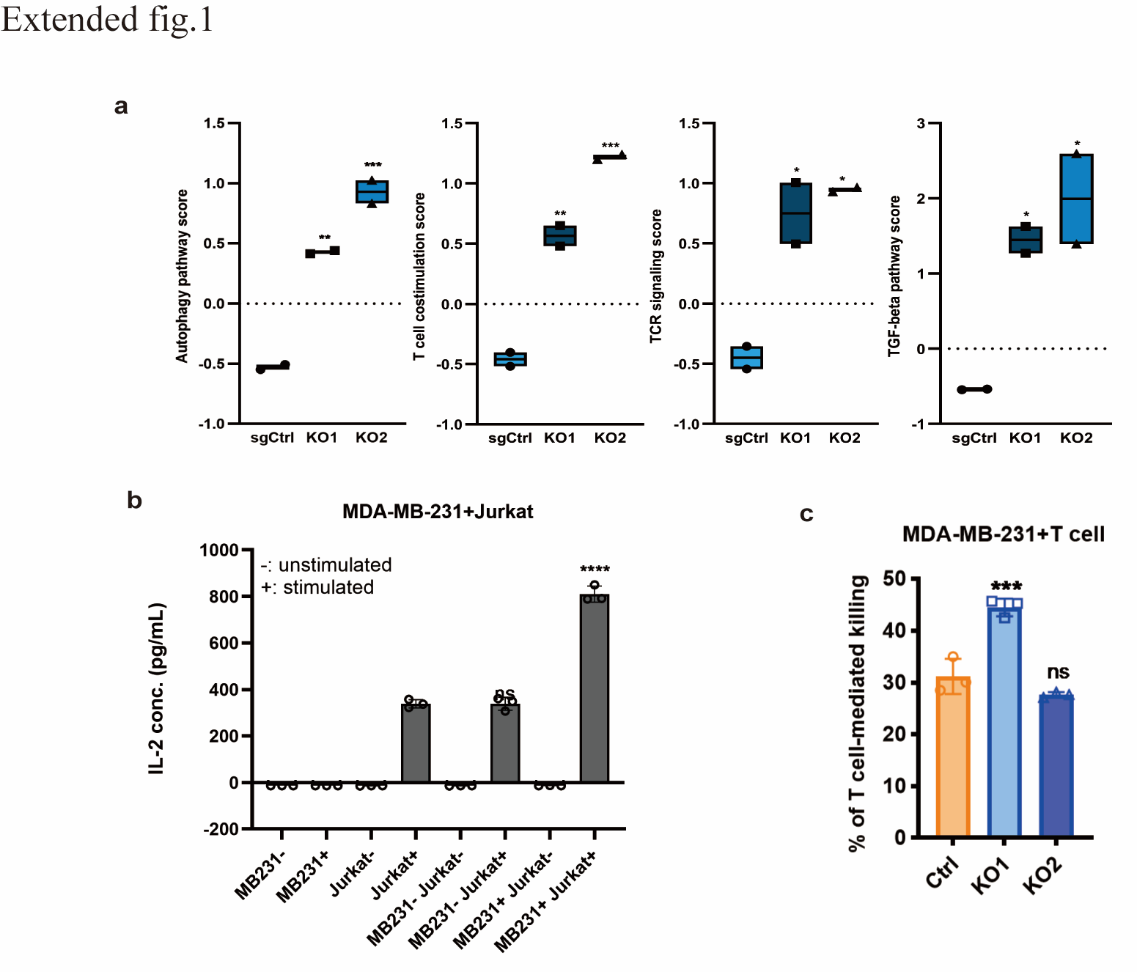
**

**Supplementary figure 1**: a. Multiple pathway scores of Nanostring gene set analysis in MDA-MB-231 sgControl and HuR knockouts (n=2). Dots represent individual samples. b. ELISA detection of IL-2 production in the co-culture system of MDA-MB-231 and Jurkat cells (n=3). c. T cell-mediated killing ability in the co-culture system using activated T cells and MDA-MB-231 sgCtrl or HuR knockout clones (n=3). Data are presented as mean ± SEM. Two-way ANOVA (b, c). n.s.: no significance, **p* < 0.05, ***p* < 0.01, ****p* < 0.001, *****p* < 0.0001.


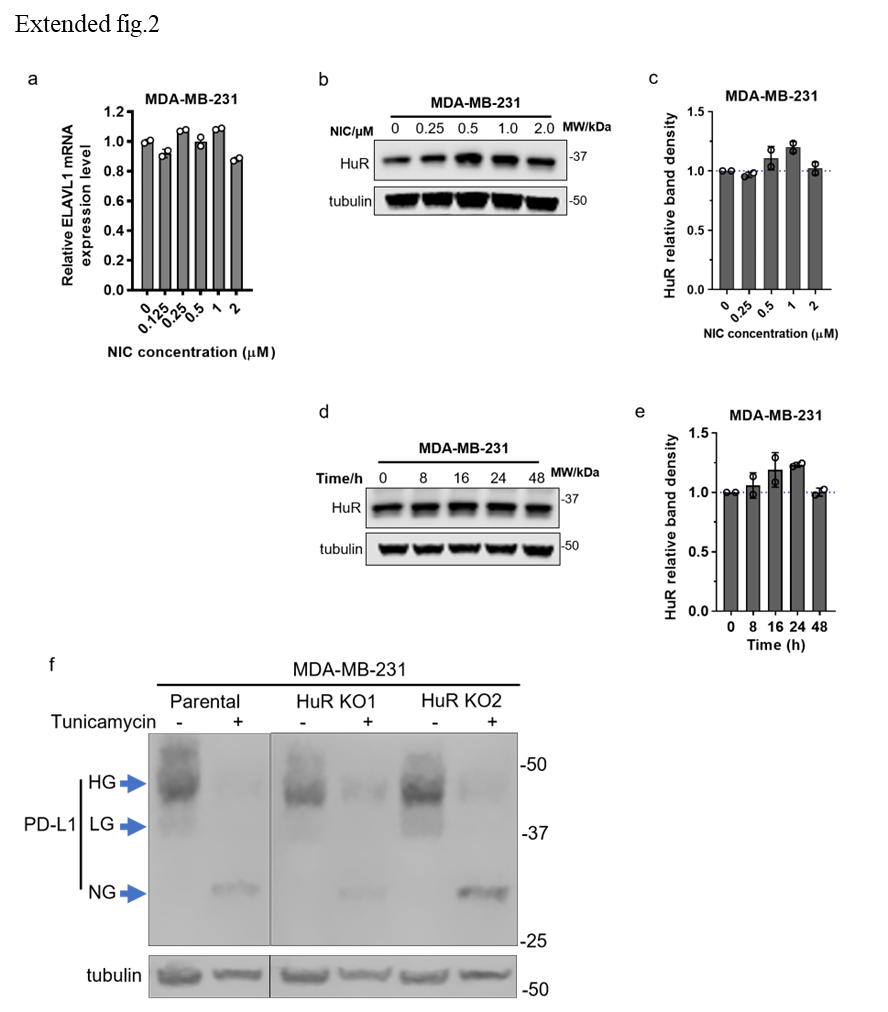


**Supplementary figure 2**: a. HuR mRNA (*ELAVL1*) expression in MDA-MB-231 cells treated with different concentrations of niclosamide for 24 hours (n=2). b-c. Representative Western blot of HuR protein levels in MDA-MB-231 treated with different concentrations of niclosamide for 48 hours (b) and the quantification of HuR band intensity (c). d-e. Representative Western blot of HuR protein levels in MDA-MB-231 treated with 1 µM niclosamide for different time intervals (d), and the quantification of HuR band intensity (e). f. Glycosylation pattern of PD-L1 proteins in MDA-MB-231. Cell lysates were treated with tunicamycin and analyzed by Western blot analysis. Data are presented as mean ± SEM. Two-way ANOVA (a). n.s.: no significance, **p* < 0.05, ***p* < 0.01, ****p* < 0.001, *****p* < 0.0001.


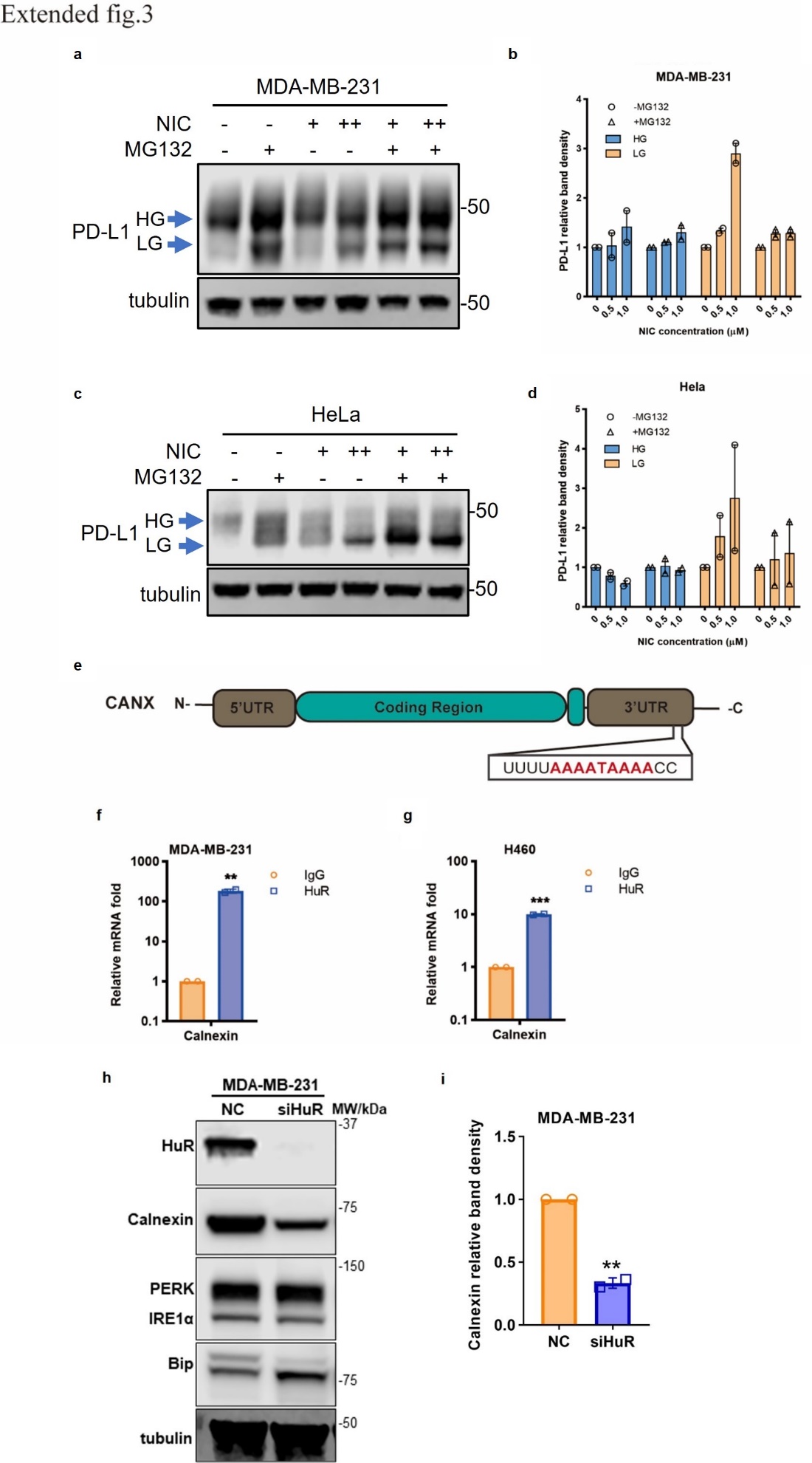


Supplementary figure 3: a-b. Representative Western blot of PD-L1 protein levels(a) and quantification(b) of PD-L1 band density in MDA-MB-231 with the treatment of 10 μM MG-132 for 16 hours. c-d. Representative Western blot of PD-L1 protein levels (c) and quantification (d) of PD-L1 band density in HeLa with the treatment of 10 μM MG-132 for 16 hours (n=2). e. The schematic of *Calnexin* mRNA. Potential AREs sequences that might bind to HuR are underlined in red. f-g. RNP-IP analysis of relative enrichment of *PD-L1* transcripts in HuR-immunoprecipitation in MDA-MB-231 (f) and H460 (g) cells. h. Representative Western blot of Calnexin and HuR protein levels in MDA-MB-231 negative control (NC) and HuR siRNA knockdown (siHuR) cells. i. Quantification of calnexin protein band intensity in control and HuR knockdown cells (n=2). Data are presented as mean ± SEM. Two-way ANOVA (f, g, i). n.s.: no significance, **p* < 0.05, ***p* < 0.01, ****p* < 0.001, *****p* < 0.0001.


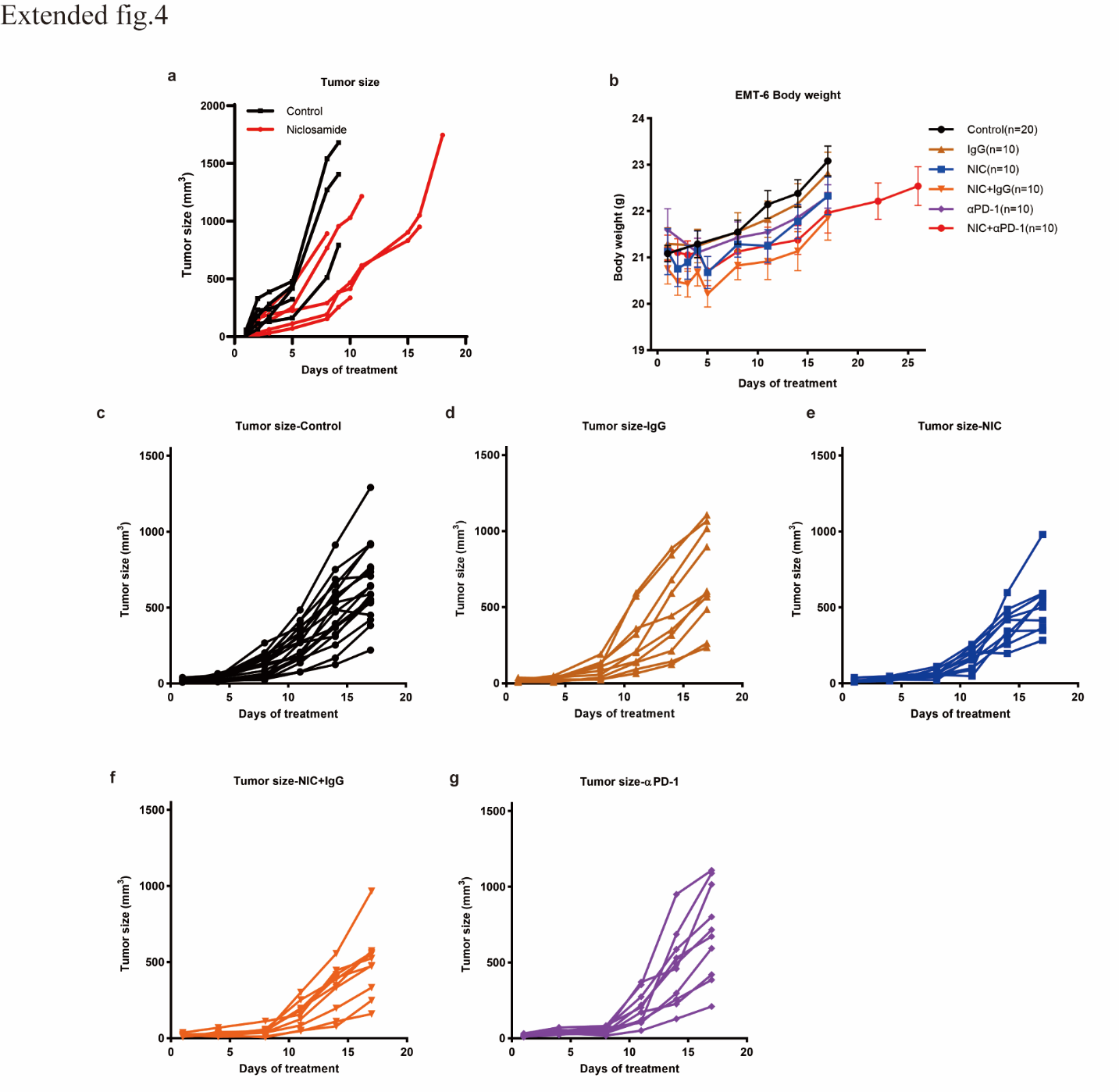


Supplementary figure 4: a. Tumor size changes in mice bearing LL/2 tumors. b. Body weight change of mice bearing EMT-6 tumors. c-g. individual tumor size of mice in the control group (c), IgG group (d), NIC group (e), NIC + IgG group (f), and PD-1 antibody group (g).


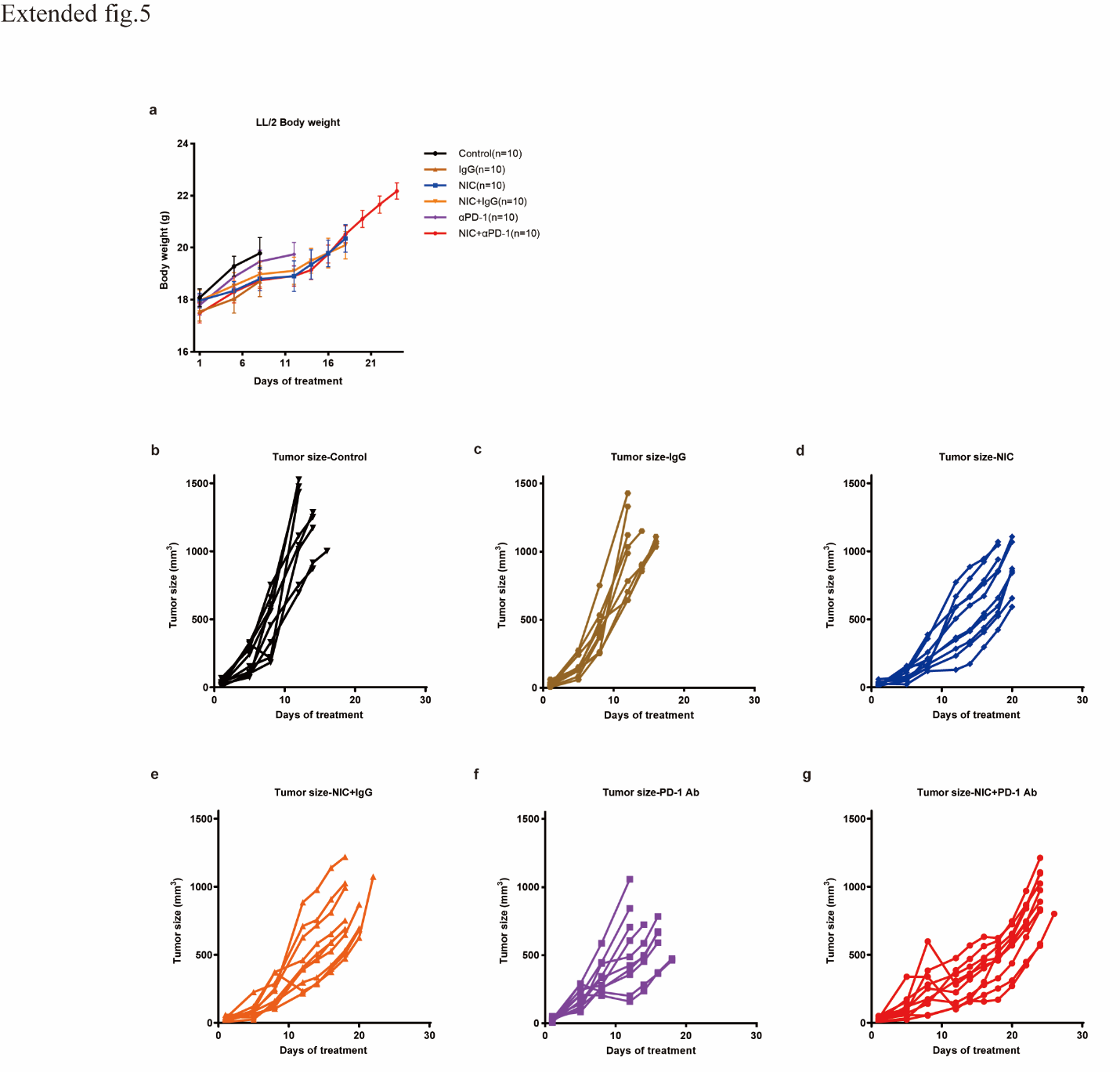


**Supplementary figure 5:** a. Body weight changes in mice bearing LL/2 tumors in different treatment groups. b-g. individual tumor size of mice in the control group (b), IgG group (c), NIC group (d), NIC + IgG group (e), PD-1 antibody group (f), and NIC + PD-1 antibody (g).

Original, uncropped Western blots


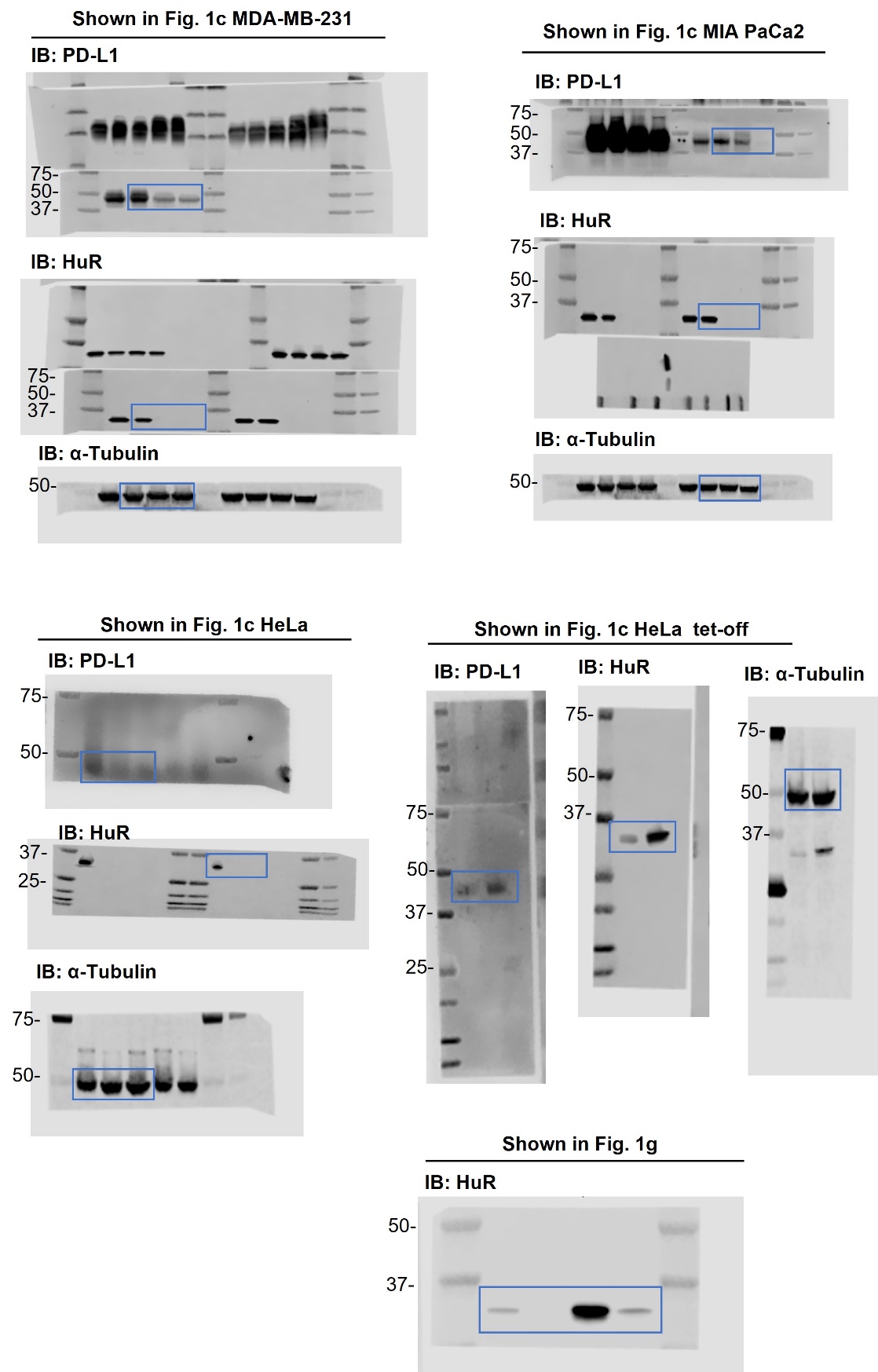
(The Western blots were imaged by LI-COR Odyssey Fc Imager)


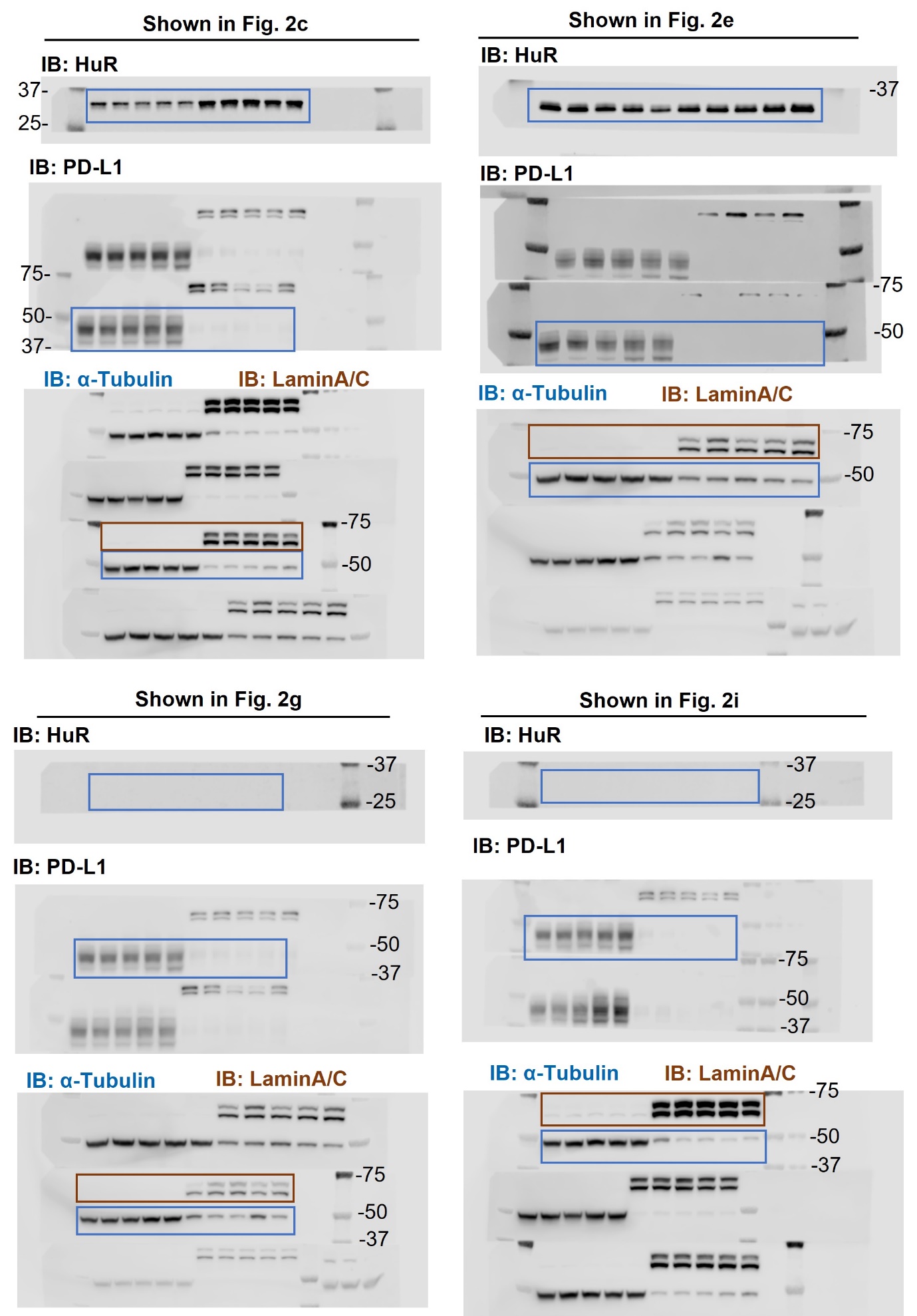


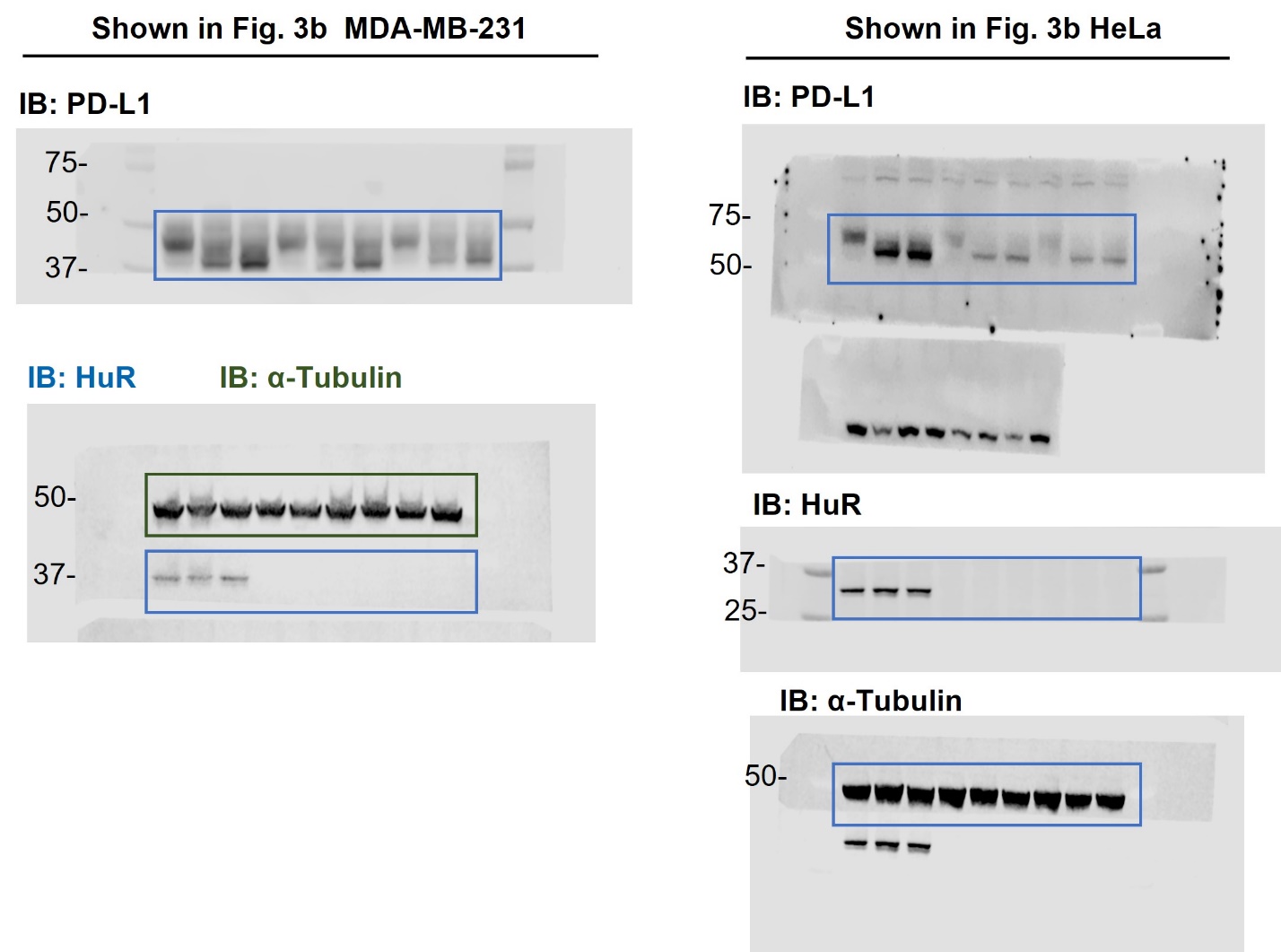


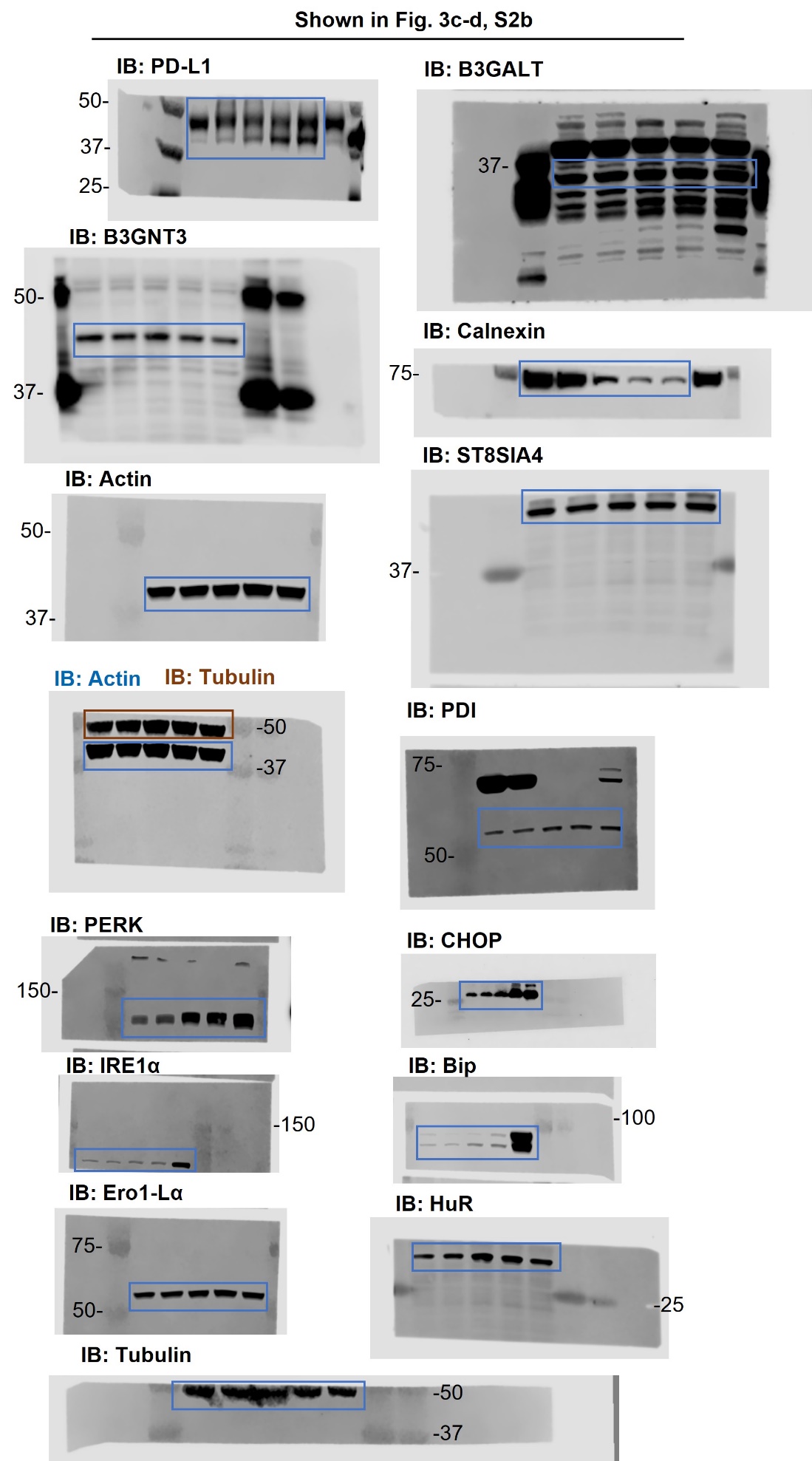


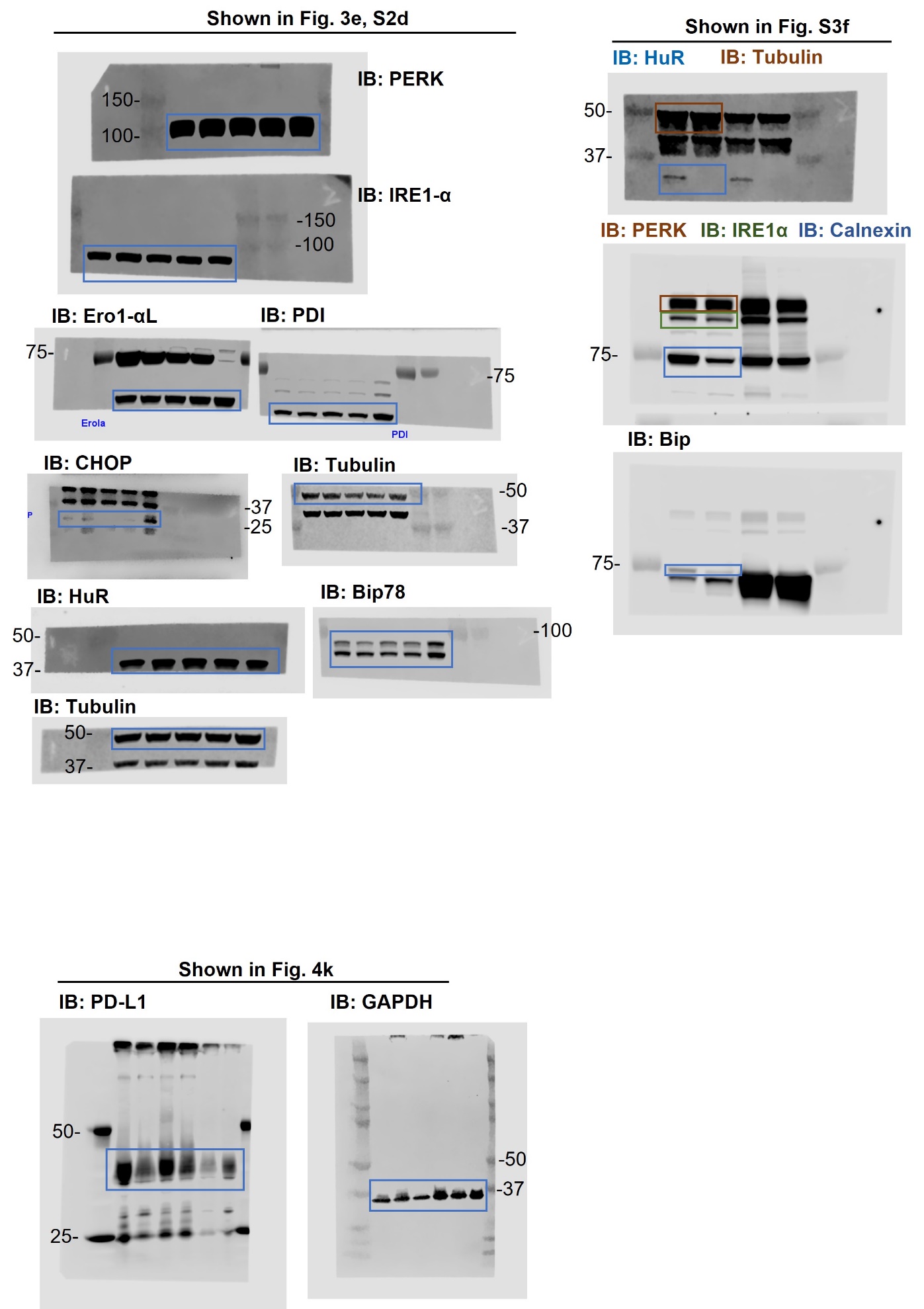


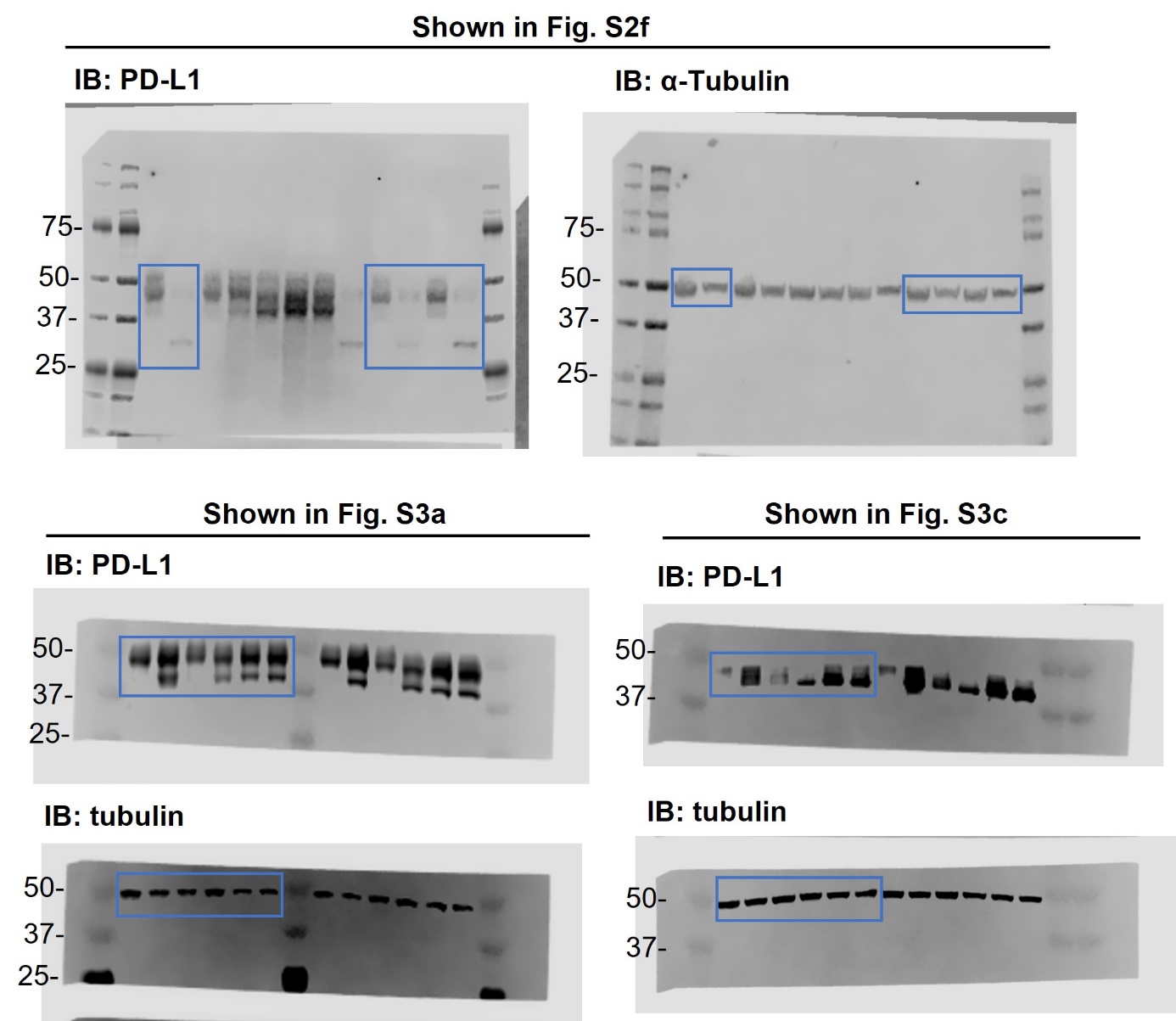


Supplementary Figure 6: Uncropped immunoblots for Fig. 1cg, Fig. 2cegi, Fig. 3b-e, Fig. 4k, Supplementary Fig. 2bdf, and Supplementary Fig. 3ach.

**Supplementary Table S1. The information on antibodies and reagents**

| **Antibodies** | | | |
| --- | --- | --- | --- |
| **Name** | **Company** | **Clone/Cat. Number** | **Application and Dilution** |
| Anti-PD-L1 | Cell Signaling Technology | E1L3N XP, Cat# 13684 | WB 1:1000 |
| Anti-Calnexin | Cell Signaling Technology | C5C9, Cat# 2679 | WB 1:1000 |
| Anti-PD-L1 | Proteintech | 2B11D11, Cat#66248 | WB: 1:1000 |
| Anti-GAPDH | Cell Signaling Technology | D16H11, Cat# 5174 | WB 1:1000 |
| Mouse anti-HuR | Santa Cruz | 3A2, Cat# sc-5621 | WB 1:500; IHC 1:50; ICC 1:200 |
| Mouse anti-GAPDH | Santa Cruz | 10B8, Cat#sc-51905 | WB 1:500 |
| Anti-α-Tubulin | Sigma-Aldrich | Cat# T5168 | WB 1:2000 |
| Goat anti-mouse IRDye secondary antibodies | LI-COR Biosciences | Cat# 926-68070 and Cat# 926-32210 | WB 1:10000 |
| Goat anti-rabbit IRDye secondary antibodies | LI-COR Biosciences | Cat# 926-68071 and Cat# 926-32211 | WB 1:10000 |
| ER Stress Antibody Sampler Kit | Cell Signaling Technology | Cat# 9956 | WB 1:1000 |
| Anti-Human CD3 antibody | Invitrogen | Cat# 16-0037-85 | T cell activation, 100 ng/mL |
| Human IgG4 (S228P) | Bio X cell | Cat# CP147 | MLR, 10 μg/mL |
| Anti-human PD-1 (Pembrolizumab) | Bio X cell | Cat# SIM0010 | MLR, 10 μg/mL |
| IgG from rat serum | Sigma-Aldrich | Cat# 14131 | Animal i.p injection, 100 µg/injection |
| I*nVivo*Plus anti-mouse PD-1 (CD279) | Bio X cell | Clone 29F. 1A12, Cat# bp0273 | Animal i.p injection, 100 µg/injection |

(WB: Western blot; IHC: Immunohistochemistry; ICC: Immunocytochemistry; MLR: Mixed lymphocyte reaction)

| **Chemicals, reagents, and kits** | | |
| --- | --- | --- |
| **Name** | **Company** | **Catalog number** |
| niclosamide | Calbiochem | Cat# 481909 |
| dimethyl sulfoxide (DMSO) | Sigma-Aldrich | Cat# D8418 |
| Tween-80 | Sigma-Aldrich | Cat# P4780 |
| HuR siRNA smart pool | Horizon | \ |
| Triton X-100 | Sigma-Aldrich | Cat# T8787 |
| Bovine serum albumin (BSA) | Fisher Scientific | Cat# BP1605-100 |
| glycine | Sigma-Aldrich | Cat# G7126 |
| Actinomycin D | Sigma-Aldrich | Cat# A1410 |
| SlowFade Gold antifade reagent | Invitrogen | Cat# S36938 |
| Clarity Western ECL substrate kit | Bio-Rad | Cat# 1705060 |
| DAPI containing mounting medium | Vector Laboratories | Cat# H-1200 |
| phorbol myristyl acetate (PMA) | Sigma-Aldrich | CAS 16561-29-8, Cat# P1585 |
| ionomycin | Sigma-Aldrich | CAS 56092-81-0 |
| ImmunoCult^TM^-XF T Cell Expansion Medium | STEMCELL Technologies | Cat# 10981 |
| ImmunoCult^TM^ Human CD3/CD28/CD2 T cell activator | STEMCELL Technologies | Cat# 10970 |
| Human Recombinant IL-2, ACF | STEMCELL Technologies | Cat# 78145 |
| *InVivo*Pure^TM^ pH 7.0 Dilution Buffer | Bio X cell | Cat# IP0070 |
| Lipofectamine 3000 transfection kit | Invitrogen | Cat# L3000-015 |
| NE-PER Nuclear and cytoplasmic extraction reagents kit | Thermo Scientific | Cat# 78835 |
| ELISA MAX^TM^ Deluxe Set Human IL-2 kit | Biolegend | Cat# 431804 |

**Table S2. The information on cancer and immune cell lines**

| **Name** | **Company** | **Type** |
| --- | --- | --- |
| MDA-MB-231 | ATCC | Human breast cancer cell line |
| SUM159 | ATCC | Human breast cancer cell line |
| MIA PaCa-2 | ATCC | Human pancreatic cancer cell line |
| H460 | ATCC | Human lung cancer cell line |
| A549 | ATCC | Human lung cancer cell line |
| (HEK) 293-FT | ATCC | human embryonal kidney cell line |
| Lewis lung-2 (LL/2) | ATCC | Mouse lung cancer cell line |
| EMT6 | ATCC | Mouse breast cancer cell line |
| HeLa | Dr. Dan Dixon | Human cervical cancer cell line |
| Peripheral blood mononuclear cells (PBMCs) | STEMCELL technologies | Human peripheral blood mononuclear cells |

**Supplementary Table S3. The sequences of the oligonucleotide primers used for qPCR and IP**

| ***Application*** | ***Gene*** | ***Abbr.*** | ***Primer Sequence (5'-3')*** |
| --- | --- | --- | --- |
| qPCR | CD274 molecule | *CD274* | Forward: GGCATTTGCTGAACGCATTTACT |
|  |  |  | Reverse: TAGTGCAGCCAGGTCTAATTGT |
|  | ELAV-like RNA binding protein 1 | *ELAVL1* | Forward: GCAGCATTGGTGAAGTTGAA |
|  |  |  | Reverse: GCGGTCACGTAGTTCACAAA |
|  | Glyceraldehyde-3-phosphate  dehydrogenase | *GAPDH* | Forward: ATGTTCGTCATGGGTGTGAA |
|  |  |  | Reverse: GGTGCTAAGCAGTTGGTGGT |
|  | Transforming growth factor beta 2 | *TGFB2* | Forward: GCCTGAACAACGGATTGAGC |
|  |  |  | Reverse: ATCGAAGGAGAGCCATTCGC |
|  | Calnexin | *CANX* | Forward: CCTCTCTCTTTACTGCGGCG |
|  |  |  | Reverse: TCCAGTCTCCGCTCTAGCC |
| RNA pulldown | CD274 molecule | *CD274* | CUUAUUUAUUUUGUUACUU |
|  | Calnexin | *CANX* | UUUUAAAATAAAACC |


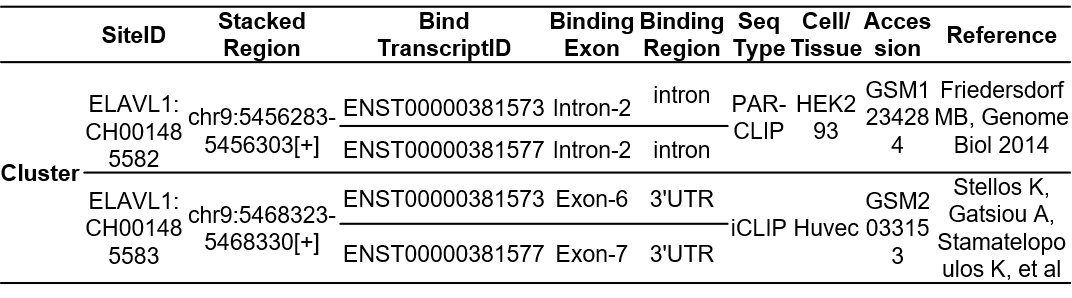
Supplementary Table S4. HuR-*CD274* interactions supported by CLIP-seq data in starBase database (starbase.sysu.edu.cn)
